# The *Aspergillus nidulans* Pyruvate Dehydrogenase Kinases Are Essential To Integrate Carbon Source Metabolism

**DOI:** 10.1534/g3.118.200411

**Published:** 2018-05-24

**Authors:** Laure Nicolas Annick Ries, Leandro José de Assis, Fernando José Santos Rodrigues, Camila Caldana, Marina Campos Rocha, Iran Malavazi, Özgür Bayram, Gustavo H. Goldman

**Affiliations:** * Faculdade de Ciências Farmacêuticas de Ribeirão Preto (FCFRP), Universidade de São Paulo, CEP 14040-903, Ribeirão Preto, São Paulo, Brazil;; † Instituto de Investigação em Ciências da Vida e Saúde, Campus de Gualtar, Universidade do Minho, 4710-057, Braga, Portugal;; ‡ Laboratório Nacional de Ciência e Tecnologia do Bioetanol (CTBE), Centro Nacional de Pesquisa em Energia e Materiais (CNPEM), CEP 13083-970, Campinas, São Paulo, Brazil;; § Departamento de Genética e Evolução, Centro de Ciências Biológicas e da Saúde, Universidade Federal de São Carlos, São Carlos, São Paulo, Brazil;; ** Maynooth University, Biology Department, Maynooth, Co. Kildare, Ireland

**Keywords:** *Aspergillus nidulans*, pyruvate dehydrogenase kinases, carbon source utilization and regulation, carbon catabolite repression

## Abstract

The pyruvate dehydrogenase complex (PDH), that converts pyruvate to acetyl-coA, is regulated by pyruvate dehydrogenase kinases (PDHK) and phosphatases (PDHP) that have been shown to be important for morphology, pathogenicity and carbon source utilization in different fungal species. The aim of this study was to investigate the role played by the three PDHKs PkpA, PkpB and PkpC in carbon source utilization in the reference filamentous fungus *Aspergillus nidulans*, in order to unravel regulatory mechanisms which could prove useful for fungal biotechnological and biomedical applications. PkpA and PkpB were shown to be mitochondrial whereas PkpC localized to the mitochondria in a carbon source-dependent manner. Only PkpA was shown to regulate PDH activity. In the presence of glucose, deletion of *pkpA* and *pkpC* resulted in reduced glucose utilization, which affected carbon catabolite repression (CCR) and hydrolytic enzyme secretion, due to de-regulated glycolysis and TCA cycle enzyme activities. Furthermore, PkpC was shown to be required for the correct metabolic utilization of cellulose and acetate. PkpC negatively regulated the activity of the glyoxylate cycle enzyme isocitrate lyase (ICL), required for acetate metabolism. In summary, this study identified PDHKs important for the regulation of central carbon metabolism in the presence of different carbon sources, with effects on the secretion of biotechnologically important enzymes and carbon source-related growth. This work demonstrates how central carbon metabolism can affect a variety of fungal traits and lays a basis for further investigation into these characteristics with potential interest for different applications.

The metabolism of a given organism controls growth and development and mediates the regulation of intrinsic cellular pathways which impact other traits, such as, in the case of filamentous fungi, secondary metabolite (SM) production and enzyme secretion (Andersen 2014). Metabolism can further be distinguished into primary and specialized metabolism, where the former sustains the living state of the cell as it is responsible for maintaining growth, development and reproduction, while the latter is involved in the production of other metabolites which can have ecologically relevant functions but are not required for the immediate survival of the cell (Brakhage 2013). In *Aspergillus spp*., the regulation of SM production is of particular interest due to their potential pharmaceutical properties (Klejnstrup *et al.* 2012) and the impacts they can have on agriculture, economy and medicine (Amare and Keller 2014; Inglis *et al.* 2013). While SM production has been extensively studied, the regulation of primary metabolism in biotechnologically and medically important *Aspergillus spp*. has been largely neglected (Andersen 2014).

The use of externally available carbon sources constitutes a main branch in primary metabolism. In *A. nidulans*, primary carbon metabolism encompasses, among others, glycolysis, glycerol metabolism, fermentation, the tricarboxylic acid (TCA) cycle and gluconeogenesis (de Assis *et al.* 2015a). These processes further branch out into several other metabolic pathways which generate sugars required for fungal cell wall biosynthesis, produce the intracellular storage compounds trehalose, glycogen, and glycerol, generate precursors for nucleotide sugar synthesis via the pentose phosphate pathway (PPP) (de Assis *et al.* 2015a) as well as precursors for amino acid biosynthesis and lipid storage (Hynes *et al.* 2006; Shimizu *et al.* 2010; Costenoble *et al.* 2011). The aforementioned biosynthetic pathways are essential for fungal survival as they mediate the adaptation to the extracellular environment, protect against external stresses and are important for growth and development (Abad *et al.* 2010; Al-Bader *et al.* 2010; Hondmann *et al.* 1991). Most filamentous fungi are able to metabolize a wide range of different carbon sources, but the preferred sugar is glucose which provides quick energy for growth and niche colonization (Ruijter and Visser 1997). The selection of the energetically most favorable carbon source is known as carbon catabolite repression (CCR), a mechanism which prevents the expression of genes required for the utilization of alternative carbon sources. Although the mechanism of CCR has been investigated in detail in several filamentous fungi (Ries *et al.* 2016; Niu *et al.* 2015; Antoniêto *et al.* 2016; Sun and Glass 2016), carbon source sensing and the accompanying signal transduction pathways remain largely uncharacterized.

The pyruvate dehydrogenase complex (PDH) is a multi-enzyme complex which is crucial for carbon metabolism as it links glycolysis to the TCA cycle by catalyzing the decarboxylation of pyruvate to acetyl-coA (Kolobova *et al.* 2001). The PDH acts as a metabolic switch, regulating the use of alternative carbon sources through controlling the flux of pyruvate to respiration or preserving it for gluconeogenesis (de Assis *et al.* 2015a; Wu *et al.* 2001). The PDH is composed of three catalytic enzymes: pyruvate dehydrogenase (E1), dihydrolipoamide acetyltransferase (E2) and dihydrolipamide dehydrogenase (E3) (Gao *et al.* 2016; Patel and Korotchkina 2006). The mammalian PDH further contains bound pyruvate dehydrogenase kinases (PDHK) and phosphatases (PDHP) which regulate the activity of the catalytic subunits by phosphorylation (de-activation) and de-phosphorylation (activation). In mammalian cells, 50% of the daily calorie uptake passes through the PDH and this rate-limiting, flux-generating metabolic reaction therefore needs to be tightly regulated (Patel and Korotchkina 2006). Mammalian PDH is targeted by phosphorylation on three different sites in the α-subunit of E1 by four isoforms of PDHK (Kolobova *et al.* 2001; Patel and Korotchkina 2006). The four PDHKs differ in their specificity for each of the three phosphorylation sites and are expressed in a tissue-dependent manner (Patel and Korotchkina 2006). Similarly, the two mammalian PDHP isoforms can de-phosphorylate all three PDH sites with different affinities and are localized in a tissue-specific manner (Patel and Korotchkina 2006). The activity of the PDH is dependent on cellular pyruvate (substrate) concentration-dependent signals as well as the energetic state of the cell (Bao *et al.* 2004a; Bao *et al.* 2004b). Pyruvate allosterically regulates PDHKs, with high levels resulting in PDHK inhibition and subsequently in PDH activation. Furthermore, high levels of ADP, NAD^+^, CoA and P_i_ signal energy depletion and require a de-activation of the PDH (Bao *et al.* 2004a; Bao *et al.* 2004b) with the PDHPs requiring Ca^2+^ and Mg^2+^ as cofactors for catalysis (Patel and Korotchkina 2006).

In *S. cerevisiae*, the PDH is regulated by the combined action of two non-bound PDHKs and PDHPs which target only one phosphorylation site in the E1 α-subunit of the PDH (Gey *et al.* 2008; Krause-Buchholz *et al.* 2006; Steensma *et al.* 2008). Similarly, the PDH of *Neurospora crassa* was shown to be subjected to phosphorylation and de-phosphorylation, which is thought to also occur on one site in the E1 α-subunit (Patel and Korotchkina 2006; Wieland *et al.* 1972). Deletion of the two *S. cerevisiae* PDHKs, Pkp1p and Pkp2p, resulted in reduced growth on acetate and ethanol which was suggested to be due to a predicted futile carbon utilization cycle (Steensma *et al.* 2008). Deletion of the *Fusarium graminearum* PDHK, FgPDK1, caused reduced growth on minimal medium supplemented with sucrose and had an impact on fungal morphology, conidiation and pathogenicity (Gao *et al.* 2016). In *A. nidulans*, genes encoding PDH subunits were identified early on (Payton *et al.* 1977; Bos *et al.* 1981). The two PDHPs PtcD and PtcE were shown to be important for glucose-related growth, consumption and respiration as well as for maintaining intracellular pyruvate concentrations, α-ketoglutarate dehydrogenase (a TCA cycle enzyme) activity and the ADP/ATP ratio (de Assis *et al.* 2015a), whereas the PDHK PkpC (AN6207) was shown to be important for growth on cellulose and cellulase secretion (Brown *et al.* 2013) with PkpA and PkpB remaining uncharacterized.

The aim of this study was therefore to investigate the role played by the three *A. nidulans* PDHKs in carbon source-dependent growth and development, in order to uncover and understand regulatory mechanisms in carbon metabolism which could prove useful for biotechnological and biomedical applications. The functional roles of all three mitochondrial-localized PDHKs diverged in a carbon source-dependent manner, with PkpC being crucial for the utilization of preferred (glucose) and alternative (cellulose, acetate) carbon sources by regulating carbon source-specific metabolic progression and, in the case of glucose and cellulose, enzyme secretion. PkpA was shown to also be important for glucose utilization and to positively regulate PDH activity. In summary, this study reveals novel traits in fungal central metabolism and shows how a de-regulation in this process can affect various fungal traits which are considered important for biotechnological and biomedical applications.

## Materials And Methods

### Strains and media

All strains used in this study are listed in Table S1. Strains were grown in biological triplicates at 37° in liquid or solid minimal medium (MM) and complete medium (YUU) as previously described (Ries *et al.* 2016). Strain auxotrophies were complemented with the respective supplement [uridine (1.2 g/l), uracil (1.2 g/l), pyridoxine (0.005 mg/μl), urea (0.3 mg/ml; the *pkp* single deletion and *gfp*-tagged strains), sodium thiosulfate (0.63 mg/ml)], which were added to all strains at all times. Mycelia were harvested or transferred to different carbon sources as previously described (Ries *et al.* 2016).

### PCR and DNA manipulations

All primers used in this study are listed in Table S2. PCR reactions were carried out according to manufacturer’s instructions. All DNA fragments for yeast transformations were amplified with Phusion High Fidelity DNA polymerase (New England Biolabs). DNA cassettes for *A. nidulans* transformations were generated using Takara Taq DNA polymerase (TaKaRa). Strains resulting from sexually crossing the parental types were confirmed by PCR using Taq DNA polymerase (ThermoFisher).

The delta pkpA and delta pkpB strains were previously constructed (De Souza *et al.*, 2013). To generate the Δ*pkpB* strain, the *pkpB* gene was replaced with the *pyrG* marker gene in strain TN02a3. The 5′ and 3′ UTR regions of *pkpB* with *pyrG* overhangs were amplified with primers 1, 2, 3 and 4. The Δ*pkpA*, Δ*pkpB* and Δ*pkpC* strains were complemented with the respective *gfp*-tagged gene and the pyridoxine marker gene. The 5′ and 3′ UTR regions with the respective overhangs of *pkpA* were amplified with primers 5, 6, 7 and 8; of *pkpB* with primers 1, 10, 9 and 4; of *pkpC* with primers 11, 12, 13 and 14. Deletion of *pkpA*, *pkpB* and *pkpC* was confirmed with primers 15 and 16, 1 and 4 and 17 and 18 respectively. The pyridoxine marker gene was amplified from plasmid pTN1 (Nayak *et al.* 2006) with primers 19 and 20. The *gfp* and *pyrG* genes, were amplified as previously described (Ries *et al.* 2016), except that primer 21 was used to amplify *gfp*. The *creA*::*gfp* construct was confirmed by PCR as described previously (Ries *et al.* 2016). Homologous integration of DNA cassettes was confirmed by PCR and one positive candidate was chosen for subsequent analysis.

### Transformation of S. cerevisiae and gDNA extraction

To generate DNA cassettes for *A. nidulans* strain complementation or deletion, *S. cerevisiae* strain Sc9721 was transformed with plasmid pRS426 (linearized with the restriction enzymes *Bam*HI and *Eco*RI) and the respective gene fragments as previously described (Gietz and Schiestl 1991). DNA was extracted (Goldman *et al.* 2003) from positive yeast transformation colonies which were first grown in 5 ml Sc URA- liquid medium for 2 days at 30°. Yeast gDNA was checked by PCR to confirm the correct construction.

### Transformation of *A. nidulans* and gDNA extraction

*A. nidulans* transformations were carried out as previously described (Sambrook and Russell 2001). Selected colonies were purified over three rounds (Ries *et al.* 2016), gDNA was extracted (Sambrook and Russell 2001) and the construction confirmed by PCR (data not shown).

### Strain construction by crossing

The CreA::GFP Δ*pkpA*, CreA::GFP Δ*pkpB*, CreA::GFP Δ*pkpC* and the pyruvate dehydrogenase kinase double deletion strains were generated by sexually crossing the parental types as previously described (Ries *et al.* 2016). To get the protein kinase double deletion strains, the Δ*pkpA*, Δ*pkpB* and Δ*pkpC* strains (pyridoxine auxotrophs) were first crossed with strain R21 (Table S1) in order to generate the respective protein kinase deletion strains which were auxotrophic for PABA (para aminobenzoic acid) but not for pyridoxine. Gene deletions in the PABA auxotrophic candidates were confirmed by PCR (as described above). Then, the respective single protein kinase deletion strains were crossed with each other (PABA auxotroph with pyridoxine auxotroph) in order to generate the double deletion strains. All strains were confirmed by PCR (as described above) for homologous gene deletion and two positive candidates were selected for further analysis.

### RNA extraction, cDNA synthesis and RT-qPCR

Mycelial RNA extraction and purification of biological triplicates were carried out as described previously (Ries *et al.* 2016). cDNA was synthesized from 1 μg RNA using the Superscript III Reverse Transcriptase kit (Invitrogen) according to manufacturer’s instructions. RT-qPCR reactions of the biological triplicates were carried out in technical duplicates as previously described (Ries *et al.* 2016). Briefly, 20 μl reactions containing 1 μl cDNA or 1 μl of known standard curve DNA, 10 μl SYBR Green PCR Master Mix (AB Applied Biosystems) and 15 pmol/μl forward and reverse primers were carried out in technical triplicates in the 7500 Fast Real-Time PCR thermocycler and gene expression by absolute quantification was analyzed using the 7500 Fast system v.1.4.0 (AB Applied Biosystems).

Primer pairs 22 and 23, 24 and 25 and 26 and 27 were used for *pkpA*, *pkpB* and *pkpC* amplification (S2 Table). The glucose transporter-encoding genes *hxtB*, was amplified with primer pairs 28 and 29 (S2 Table).

### Microscopy

Biological triplicates of strains to be analyzed by microscopy were inoculated in 5 ml MM supplemented with the respective carbon source at 25° as described previously (Ries *et al.* 2016). Nuclei were stained with 1 μg/ml Hoechst 33342 (Life Technologies) at RT for 5 min.

To assess spore swelling and germ tube emergence, 30 ml MM supplemented with glucose was inoculated with 10^6^ spores/ml at 37° for 8 h, 160 rpm in biological triplicates. Every 2 h, spores were spun down at 4000 rpm for 5 min at RT and viewed under the microscope. For the control condition (0 h), spore size was assessed without inoculation in glucose-containing MM.

Conidia and mycelia were viewed under a Carl Zeiss (Jena, Germany) AxioObserver.Z1 fluorescent microscope as described previously (Ries *et al.* 2016). Hoechst-stained hyphae were viewed with the same light spectrum that is used for DAPI staining (Ries *et al.* 2016). Nuclei were counted and the percentage of CreA::GFP-containing nuclei was calculated. Spore sizes were measured using the AxioVision software (version 3.1).

### Phylogenetic tree construction

Protein sequences of PkpA, PkpB and PkpC from *A. nidulans*, *A. fumigatus*, *A. niger*, *A. terreus*, *A. flavus* were retrieved from www.aspgd.org. The protein sequences of the homologs in other fungi were obtained by carrying out a BLAST search in the respective database [*S. cerevisiae* (www.yeastgenome.org), *Schizosaccharomyces pombe* (www.pombase.org), *Candida albicans* (www.candidagenome.org), *C. glabatra* (www.candidagenome.org)]. For *Penicillium chrysogenum*, *Neurospora crassa*, *Trichoderma reesei*, *T. atroviride*, *T. harzianum*, *T. virens* and *Fusarium oxysporum* the respective database from the JGI (Joint Genome institute) Genome portal (http://genome.jgi.doe.gov/) was used. All protein sequences were uploaded into the Phylogeny.fr “One click” mode (www.phylogeny.fr) which constructed the phylogenetic tree (Dereeper *et al.* 2010; Dereeper *et al.* 2008). This program is run with the aLRT (approximate Likelihood Ratio Test) statistical test of branch support (Anisimova and Gascuel 2006).

The alignment of PkpA, PkpB and PkpC was done using ClustalW multiple sequence alignment (http://www.genome.jp/tools/clustalw/) and domain analysis was carried out using SMART (http://smart.embl-heidelberg.de/smart/set_mode.cgi?NORMAL=1) (Schultz *et al.* 1998; Letunic *et al.* 2015).

### Determination of extracellular glucose concentrations

Extracellular glucose concentrations in the culture supernatants of biological triplicates were measured using the Glucose GOD-PAP Liquid Stable Mono-reagent kit (LaborLab Laboratories Ltd. Guarulhos, São Paulo, Brazil) according to the manufacturer’s instructions.

### Determination of extracellular protein secretion and cellulase and xylanase activities

To assess total extracellular protein secretion, mycelia grown from an equal amount of spores of biological triplicates were separated from the culture supernatants by miracloth filtering. Supernatants (20 ml per sample) were first frozen at -80° before they were freeze-dried and subsequently re-suspended in 2 ml buffer (50 mM Tris-HCl pH 7.0, 50 mM NaF, 1 mM DTT, 1 mM Na_3_VO_4_, 1 μg/ml aprotinin, 1 μg/ml pepstatin, 1 μg/ml leupeptin, 1 mM PMSF). Next, 20 μl of the freeze-dried, re-suspended samples were prepared for gel analysis according to manufacturer’s instructions (Bolt Mini Gels, Life Technologies). Samples were centrifuged for 1 min at room temperature (RT) at maximum speed. Denatured samples (20 μl) were then run on a pre-made gel (BoltMini Gels, Life Technologies) at 150 V for 1 h. Gels were subsequently silver-stained according to (Blum *et al.* 1987).

Endo-xylanase and cellulase activities were measured exactly as described in (Ries *et al.* 2016).

### Pyruvate and metabolic enzyme measurements

Pyruvate concentrations and all enzyme activities were measured in technical duplicates from biological triplicates. Pyruvate concentrations and alpha-ketoglutarate dehydrogenase (KGDH) activity in mycelia was measured as previously described (de Assis *et al.* 2015a). Briefly, total cell lysates were prepared and the corresponding protein concentration measured using a Bradford assay (BioRad, according to manufacturer’s instructions). Pyruvate levels were measured by converting pyruvate to lactate using lactate dehydrogenase which oxidizes NADH; the corresponding decline in absorbance was measured at 340 nm (Chretien *et al.* 1995). Similarly, KGDH activity was measured in 30 μg of total cellular protein (Tretter and Adam-Vizi 2004; Habelhah *et al.* 2004), and the absorbance of the reduced form of NADH was measured at 340 nm.

Alcohol dehydrogenase (ADH), pyruvate dehydrogenase (PDH) and hexokinase (HXK) activities were determined according to (Creaser *et al.* 1985; Chretien *et al.* 1995; Fleck and Brock 2010) with modifications. Proteins were extracted from ground mycelial powder by re-suspension in buffer [50 mM Tris-HCl, 50 mM NaF, 1 mM DTT (dithiothreitol), 1 mM Na_3_VO_4_, 1mM PMSF (phenylmethylsulfonyl fluoride), 1.5 mM benzamidine, 1 μg/ml aprotinin, pepstatin and leupeptin] on ice. Samples were centrifuged for 5 min at 4° before the protein concentration was measured in the supernatants by Bradford assay as described above. For ICL activity, 195 μl of buffer (250 mM potassium phosphate buffer pH 7.0, 100 mM MgCl2, 100 mM Cysteine, 100 mM Phenylhydrazine, 100 mM Isocitric acid) per sample were incubated at 37° for 10 min before 5 μg of total cell protein extract was added and enzyme activity was read at 324 nm over a time period of 15 min at 37°. For PDH activity, a total of 60 μg of protein was mixed with 180 μl of buffer (10 mM KH_2_PO_4_ pH 7.8, 10 mM KCl, 5 mM MgCl_2_, 0.5 mM NAD^+^, 200 μM coenzyme A, 200 μM TPP, 0.1% Triton X-100) and incubated at 37° for 10 min. Reaction was started by adding 1 mM pyruvate and the absorbance was measured at 340 nm over a time period of 15 min at 37°. For HXK activity, 5 μg of protein was mixed with 180 μl reaction buffer (50 mM HEPES pH 7.5, 50 mM KCl, 5 mM MgCl_2_, 2 mM ATP, 1 mM phosphoenolpyruvate, 0.4 mM NADH, 5 U/reaction pyruvate kinase, 15 U/reaction lactate dehydrogenase) and the activity was read at 340 nm during 25 min at 37° (initial slope). The reaction was started by adding 100 mM glucose and the activity was measured at 340 nm during 15 min at 37° (final slope). Activities were calculated using the determined slopes of the reaction and extinction coefficient for NADH (6.22). For HXK, the slope was determined by subtracting the final slope from the initial slope.

Protein extraction buffer served as a negative control for all enzyme activities.

### Metabolite analysis

Metabolites were extracted from 5 mg of dry-frozen, mycelial powder of four biological replicates with 1 ml of MTBE (methyl tert-butyl ether): methanol:water in a 3:1:1 (v/v/v) ratio, as described previously (Giavalisco *et al.* 2011). 100 μl of the polar phase was dried and derivatised according to (Giavalisco *et al.* 2011). Subsequently, 1 μl of the derivatised sample was analyzed on a Combi-PAL autosampler (Agilent Technologies GmbH, Waldbronn, Germany) coupled to an Agilent 7890 gas chromatograph which in turn is coupled to a Leco Pegasus 2 time-of-flight mass spectrometer (LECO, St. Joseph, MI, USA) as described previously (Roessner *et al.* 2001). Chromatograms were exported from the Leco ChromaTOF software v. 3.25 to the R software (www.r-project.org). Peak detection, retention time alignment, and library matching were performed using Target Search R-package (Weckwerth *et al.* 2004).

Metabolites were quantified by the peak intensity of a selective mass. Metabolite peak intensities were normalized by dividing them by the respective sample dry-weight, the sum of the total ion count and the global outlier replacement as described previously (Giavalisco *et al.* 2011; Cuadros-Inostroza *et al.* 2009). Principal component analysis was performed using the pcaMethods bioconductor package (Huege *et al.* 2011; Stacklies *et al.* 2007). Statistical analysis was carried out by performing Tukey-tests when comparing all genotypes or when compared to the wild-type strain in a given condition.

### Cell fractionation and Western blotting

Mitochondria were isolated from mycelia as previously described (de Castro Pimentel Figueiredo *et al.* 2011). Strains were grown from 1 × 10^8^ conidia for 24 h at 37° in 100 ml of minimal medium supplemented with 1% (w/v) glucose before being transferred to minimal medium supplemented with 1% (w/v) acetate for 1 h. Cells were harvested and immediately frozen in liquid nitrogen and subjected to cell fractionation procedures. 50 µg of protein from each sample were run on a 12% SDS-PAGE gel and electroblotted to a PVDF membrane. To detect PkpA::GFP, PkpB::GFP and PkpC::GFP, anti-GFP antibody (G1544; Sigma) was used at a 1:1000 dilution in TBS-T containing 3% skimmed milk. To detect cytoplasm, anti *S. cerevisiae* 3-Phosphoglyceric phosphokinase (Pgk1) (NE130/7S; Nordic-Immunology) was used at a 1:3000 dilution in TBS-T containing 5% skimmed milk and anti *S. cerevisiae* cytochrome C was used at a 1:1000 dilution in TBS-T containing 3% skimmed milk to confirm mitochondrial fractions. Anti-Pgk1 recognizes a single band (about 44.9 kD) corresponding to *A. nidulans* PgkA (AN1246) in the cytosolic crude extract while cytochrome C recognizes a single band (14.0 kD) corresponding to *A. nidulans* CyA gene (AN6246) in the mitochondrial extract (de Castro Pimentel Figueiredo *et al.* 2011)) Vödisch *et al.* 2009). Anti *S. cerevisiae* cytochrome C was kindly provided Dr. Mario Henrique de Barros (ICB-USP, Brazil).

All primary antibody incubations were performed at 4° for 16 h before the HRP-conjugated secondary antibody raised in rabbits (A0545; Sigma) was added at a 1:3000 dilution in TBS-T for 2 h at room temperature. Images were generated by exposing the PVDF membranes to the ChemiDoc XRS gel imaging system (BioRad).

### Protein-protein interactions

#### Protein extraction:

Crude protein extracts from mycelia were obtained by extraction at 4° from ground mycelia of biological duplicates with B250 buffer (250 mM NaCl, 100 mM Tris-HCl pH 7.5, 10% glycerol, 1Mm EDTA and 0.1% NP-40) supplemented with 1.5 ml/l of 1 M DTT, 2 pills/100 m Complete-mini Protease Inhibitor Cocktail EDTA-free (Roche), 3 ml/l of 0.5 M Benzamidine, 10 ml/l phosphatase inhibitors 100x (10 M NaF, 5 M Na_3_VO_4_, 8 M β-glycerol phosphate) and 10 ml/l 100 mM PMSF. Samples were centrifuged for 10 min at 14,000 x *g* before supernatants were collected and used for immunoprecipitation.

#### Immunoprecipitation (IP):

The total protein lysate from 4 g of ground mycelia (re-suspended in 4 ml buffer B250) was incubated with 40 µL GFP Trap magnetic beads (Chromotek) for 4 h at 4°. Beads were collected on magnetic racks and washed two times with B250 buffer without DTT and one time with B250 buffer with DTT. Immunoprecipitated samples were then digested with the “Sequencing Grade Modified Trypsin” (Promega #V5117), according to manufacturer’s instructions, before samples were de-salinized using Zip-Tip (Millipore #ZTC18S096) according to the manufacturer’s instructions.

#### Protein identification by LC (liquid chromatography)-MS/MS (tandem mass spectrometry):

Trypsin-digested peptides were separated using reverse-phase liquid chromatography with an RSLCnano Ultimate 3000 system (Thermo Scientific) followed by mass identification with an Orbitrap Velos Pro mass spectrometer (Thermo Scientific). Chromatographically separated peptides were on-line ionized by nano-electrospray (nESI) using the Nanospray Flex Ion Source (Thermo Scientific) at 2.4 kV and continuously transferred into the mass spectrometer. Full scans within m/z of 300-1850 were recorded by the Orbitrap-FT analyzer at a resolution of 30.000 (using m/z 445.120025 as lock mass) with parallel data-dependent top 10 MS2-fragmentation in the LTQ Velos Pro linear ion trap. LCMS method programming and data acquisition was performed with the software XCalibur 2.2 (Thermo Scientific) and method/raw data validation with the program RawMeat 2.1 (Vast Scientific).

MS/MS2 data processing for protein analysis and identification was done with either MaxQuant quantitative proteomic software in conjunction with Perseus software for statistical analysis or the Proteome Discoverer 1.3 (PD, Thermo Scientific) and the Discoverer Daemon 1.3 (Thermo Scientific) software using the Sequest (and/or Mascot) peptide analysis algorithm(s) and organism-specific taxon-defined protein databases extended by the most common contaminants. Proteins identified for both the wild-type (control, non-GFP-tagged) and GFP-tagged stains were subtracted from the GFP-tagged strain dataset for each condition. Experiments were carried out in biological duplicates for the control and PkpC::GFP strains for each time point and at least 2 unique peptides identified per protein were applied during the analysis. PkpC was identified in all the pull down assays confirming that the IP was successful.

### Statistical analysis

A paired, equal variance student *t*-test was carried out to calculate the P-value when comparing the deletion strains with the wild-type strain (* denotes *P* < 0.05, **denotes *P* < 0.005 and ***denotes *P* < 0.0005). A two-way ANOVA test and Bonferroni post-tests were applied when comparing multiple values, *e.g.*, when comparing glucose consumption at different time points between strains.

### Data availability

Strains are available on request. Supplemental material available at Figshare: https://doi.org/10.25387/g3.6241922.

## Results

### PkpC phylogenetically clusters apart From PkpA and PkpB

To start the characterization of the three *A. nidulans* PDHKs PkpA, PkpB and PkpC, a phylogenetic relationship was first investigated. A phylogenetic tree was constructed in order to assess the relationship between the homologs of the three PDHKs in different *Aspergillus spp*., other filamentous fungi and yeast (Figure S1A). PkpC and its homologs clustered phylogenetically apart from PkpA and PkpB, as supported by PkpA, PkpB and PkpC protein alignment (Figure S1B), and their homologs, suggesting a different evolutionary origin and/or function. All three genes were expressed transcriptionally in the wild-type strain when grown for 24 h in casamino acids (CA) and then transferred to glucose (preferred carbon source)- or cellulose (alternative carbon source) -rich medium for different amounts of time (Figure S2A). The expression of *pkpA* increased significantly during the first 24 h in cellulose- but not glucose-rich conditions when compared to the CA control condition; *pkpB* was transcriptionally induced in all here tested conditions when compared to the CA condition; whereas the expression of *pkpC* significantly increased after prolonged incubation (8 h and 16 h) in glucose and (48 h) cellulose when compared to the CA control condition (Figure S2A). These results indicate that all three PDHK-encoding genes are induced during growth on these carbon sources (Figure S2A).

### PkpC is important for growth on a wide range of carbon sources

**Figure 1:**
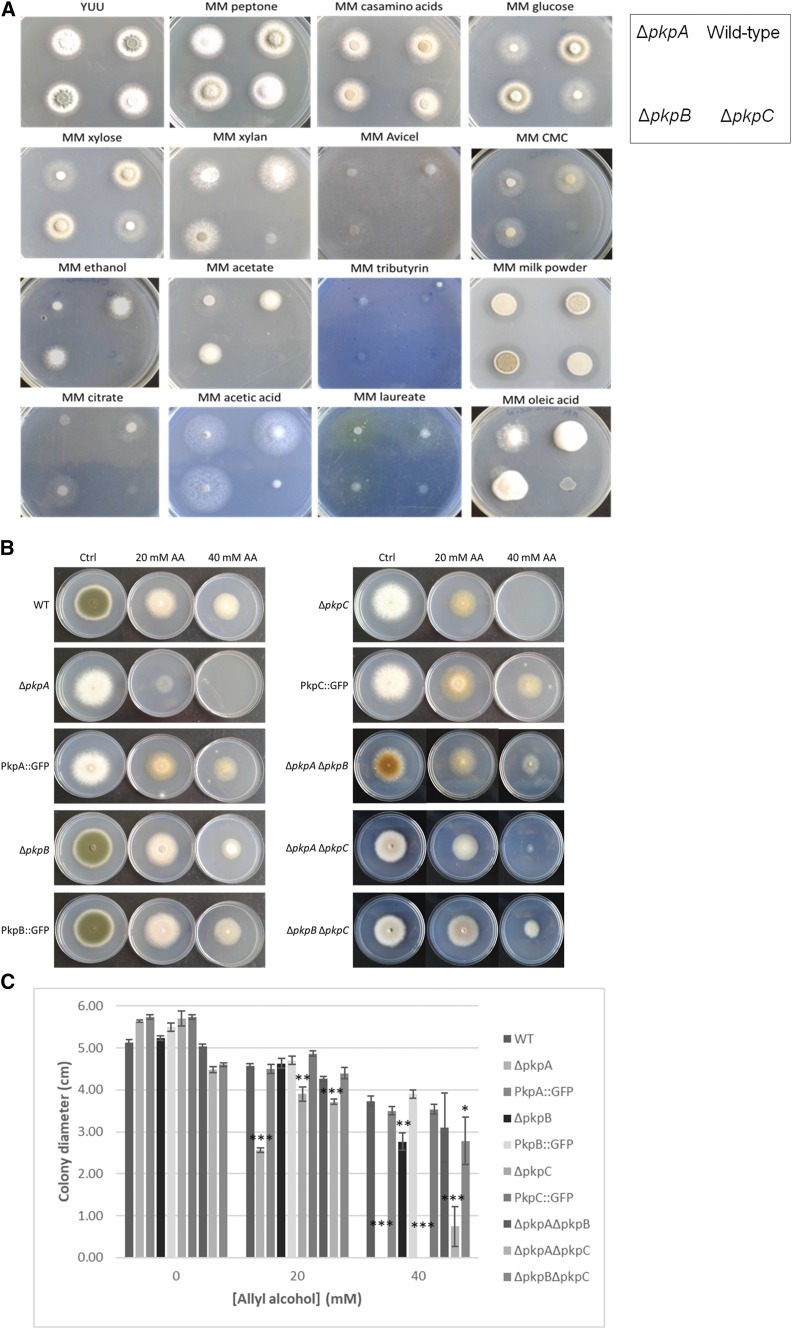
PkpC is important for growth on various carbon sources. A. The wild-type (TN02a3), Δ*pkpA*, Δ*pkpB* and Δ*pkpC* strains were grown on complete medium (YUU) or minimal medium supplemented with 1% (w/v or v/v) of all carbon sources except for laureate (2.5 mM) and oleic acid (2.5 mM). Plates were inoculated with 10^5^ spores of each strain. B-C. Complementation of the Δ*pkpA* and Δ*pkpC* strains rescues the observed growth defect in the presence of allyl alcohol (AA). The wild-type (WT), protein kinase deletion and complemented strains as well as the double deletion strains were grown on minimal medium supplemented with glucose and increasing concentrations of AA for 5 days at 30°C before (B.) representative pictures were taken and (C.) radial diameter was measured. Error bars indicate standard deviations of biological triplicates (*P-value < 0.05; **P-value < 0.005; ***P-value < 0.0005 as determined by a one-tailed, paired student *t*-test).

Carbon sources included glucose and cellulose, as they are of interest for biotechnological applications (*e.g.*, biofuel production from plant biomass) (Dashtban *et al.* 2009; Mathew *et al.* 2008), as well as the utilization of acetate, which is considered an important carbon source available to opportunistic pathogenic fungi during infection (Jiménez-López *et al.* 2013; Borregaard and Herlin 1982; Schug *et al.* 2016). In addition, growth was tested on easily assimilated carbon sources such as complete, peptone-, milk powder- and casamino-rich medium; the biotechnological-relevant polysaccharides cellulose and xylan; and other alternative carbon sources such as ethanol, citrate and fatty acids that are predicted to be important for *Aspergillus* pathogenicity (Grahl *et al.* 2011; Hynes *et al.* 2006). The wild-type and the three PDHK null mutants were grown on the aforementioned carbon sources (Figure 1A). There was no difference between the wild-type and the Δ*pkpB* strains on all tested carbon sources (Figure 1A). Deletion of *pkpC* caused a severe growth defect on a wide variety of carbon sources including cellulose (carboxymethylcellulose and Avicel), ethanol, acetate, citrate and different fatty acids (Figure 1A). Deletion of *pkpA* also caused reduced growth on ethanol, acetate and citrate although the growth defect was less pronounced than the one for the Δ*pkpC* strain (Figure 1A). Furthermore, the Δ*pkpA* and Δ*pkpC* strains also had reduced growth in the presence of glucose (Figure 1A). To determine whether the observed inhibition of growth of the Δ*pkpC* strain in the presence of acetate was due to a pH-associated transport defect (acetate is the conjugate base of acetic acid and diffuses through the membrane into the cell), the wild-type and PDHK deletion strains were grown on minimal medium supplemented with acetate at pH 4.0 and 6.5. In contrast to the other strains, the Δ*pkpC* strain did not grow at a lower pH in the presence of acetate, indicating that the observed growth defect is not pH-dependent (Figure S2B).

The wild-type and the PDHK null mutants were then grown in the presence of glucose and different concentrations of allyl alcohol (AA) in order to assess any involvement in glucose-mediated carbon catabolite repression (CCR) (Fernandez *et al.* 2012). AA is converted to the toxic compound acrolein by alcohol dehydrogenase, which is repressed in the presence of glucose and increased sensitivity and/or resistance to this compound indicates defects in CCR. There was no difference in sensitivity or resistance to AA between the wild-type and Δ*pkpB* strains (Figure 1B). The Δ*pkpA* and Δ*pkpC* strains were more sensitive to AA when compared to the wild-type strain, with Δ*pkpA* also having increased sensitivity to this compound when compared to Δ*pkpC* (Figures 1B). These results suggest that PkpA and PkpC may be involved in glucose-mediated carbon catabolite repression (CCR).

To confirm that the above described growth defects were associated to the respective gene deletion, the Δ*pkpA-C* strains were complemented with the *gfp*-tagged genes (Table S1), placed under the control of the native promoters. Radial diameter of the complemented strains was then assessed when grown for 5 days in the presence of glucose and increasing concentrations of AA at 30° (Figure 1B, C). Complementation of the Δ*pkpA* and Δ*pkpC* strains abolished increased sensitivity to AA and restored wild-type growth (Figure 1B, C). These results suggest that the observed growth phenotypes are due to the respective gene deletion. To exclude the possibility that the observed growth defects were due to morphological changes in the mitochondrial network, the size and shape of the mitochondria was assessed by microscopy in the presence of glucose in the wild-type and Δ*pkpA-C* strains. No difference in mitochondrial shape was observed in the PDHK deletion strains when compared to the wild-type strain (data not shown).

### PkpC cellular localization is carbon source-dependent

The PDH is a mitochondrial complex and cellular localization of the three *A. nidulans* Pkps was subsequently assessed. *In silico* sequence analysis by the two different programs TargetP1.1 (Emanuelsson *et al.* 2000) and MITOPROT (Claros and Vincens 1996) predicted PkpA (TargetP1.1 = 0.923, MITOPROT = 0.9912) to be targeted to the mitochondria whereas PkpC is predicted to not be mitochondrial (TargetP1.1 = 0.450; MITOPROT = 0.4327). PkpB, on the other hand, was predicted to be targeted to the mitochondria by TargetP1.1 (0.795) but not when using MITOPROT (0.4327).

**Figure 2:**
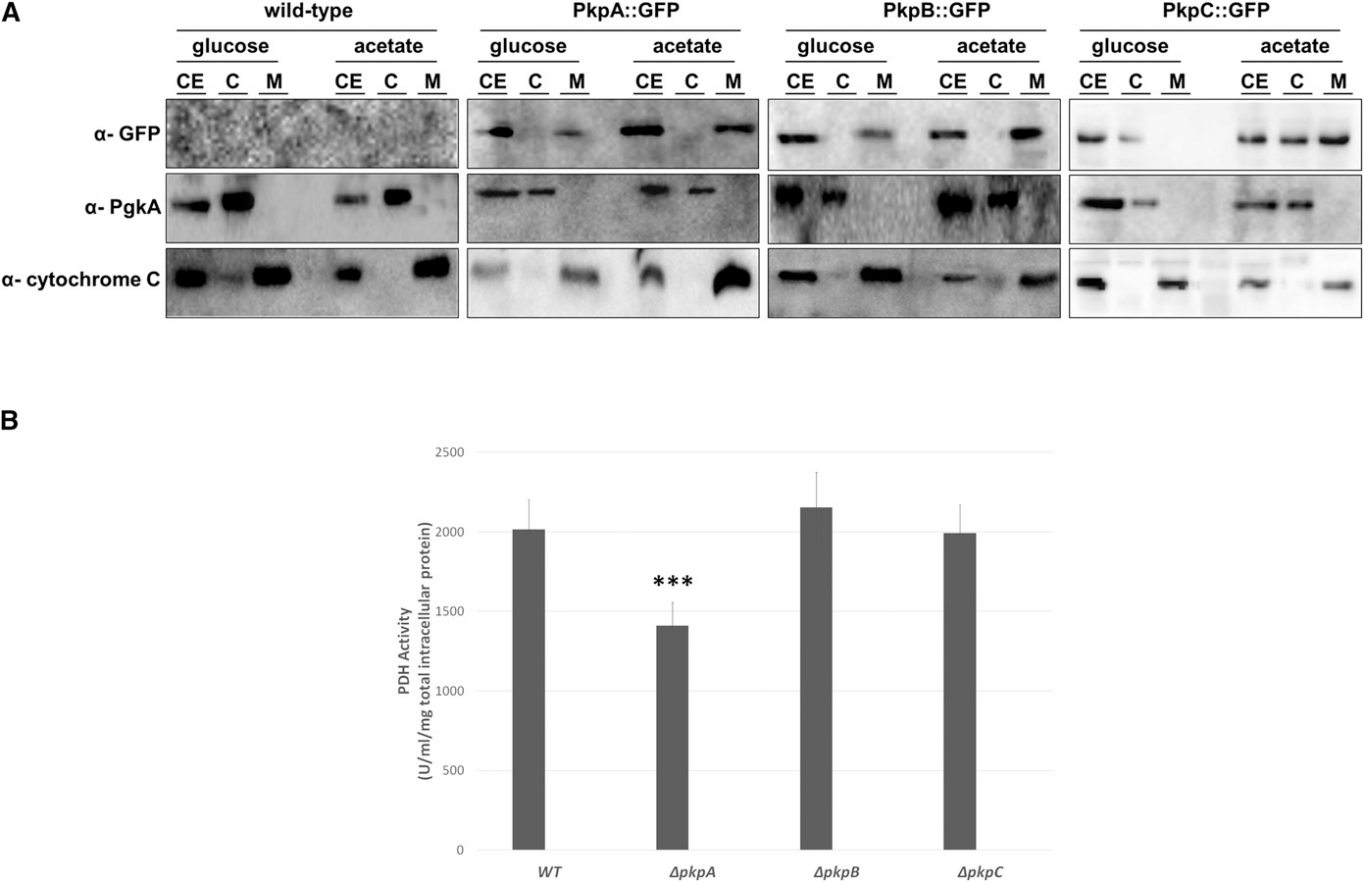
PkpC localises to the mitochondria in a carbon source-dependent manner and is not important for PDH activity. A. Western blot of the cytosolic and mitochondrial fractions of the wild-type, PkpA::GFP, PkpB::GFP and PkpC::GFP strains when grown for 24 hr in minimal medium supplemented with glucose and after transfer to acetate-rich minimal medium for 1 h. Mycelia were harvested and subjected to cell fractionation. 50 µg of protein from each cellular extract was run on a 12% SDS-PAGE gel and subsequently electroblotted to a membrane. Pkp proteins were detected by using anti-GFP antibody, whereas anti- *S. cerevisiae* Pgk1 and anti-cytochrome C antibodies were used as a fractionation controls for the cytosolic and mitochondrial enrichment, respectively (CE: crude extract, C: cytosolic extract, M: mitochondrial extract). B. Activity of the pyruvate dehydrogenase complex (PDH) in the wild-type and Δ*pkpA*, Δ*pkpB* and Δ*pkpC* strains when transferred from minimal medium supplemented with casamino acids to glucose-rich medium for 6 h. Standard deviations present the average of 3 biological replicates (***P-value < 0.0005 as determined by a one-tailed, paired student *t*-test).

To confirm the cellular localization of PkpA, PkpB and PkpC, Western blots of all three GFP-tagged proteins were carried out after cellular fractionations, on mitochondrial, cytoplasmic and total extract fractions when strains were grown for 24 h in glucose-rich medium and then transferred to minimal medium supplemented with acetate for 1 h. A non-GFP-tagged wild-type strain was used as a negative control whereas mitochondrial and cytoplasmic fractions were marked with *S. cervisiae* anti-phosphoglyceric phosphokinase (Pgk1) and anti-cytochrome C antibodies, respectively. PkpA::GFP and PkpB::GFP pre-dominantly localized to the mitochondria in the presence of glucose and acetate (Figure 2A). PkpC::GFP on the other hand, was seen in the cytoplasm and the mitochondria in the presence of acetate, whereas in the presence of glucose, it remained cytoplasmic (Figure 2A). These results suggest that PkpA and PkpB are constantly localizing with the mitochondria in all tested carbon sources, whereas PkpC is subject to mitochondrial translocation in a carbon source-dependent manner.

### PkpA positively regulates the activity of the pyruvate dehydrogenase complex (PDH)

Next, the effect of the deletion of *pkpA-C* on the activity of the PDH was determined. All strains were grown for 24 h in casamino acid-rich media and then transferred to minimal medium containing glucose for 6 h to ensure active transport and metabolism of this carbon source (Figure 2B). PDH activity was not significantly different between the wild-type (WT), Δ*pkpB* and Δ*pkpC* strains. The deletion of *pkpA* however, caused a reduction in PDH activity in the presence of glucose. These results suggest that PkpA, or a protein that is targeted by PkpA, is required for the activation of the PDH in the presence of glucose whereas deletion of the other two PDHKs had no effect on PDH activity.

### PkpA and PkpC are involved in hydrolytic enzyme secretion

**Figure 3:**
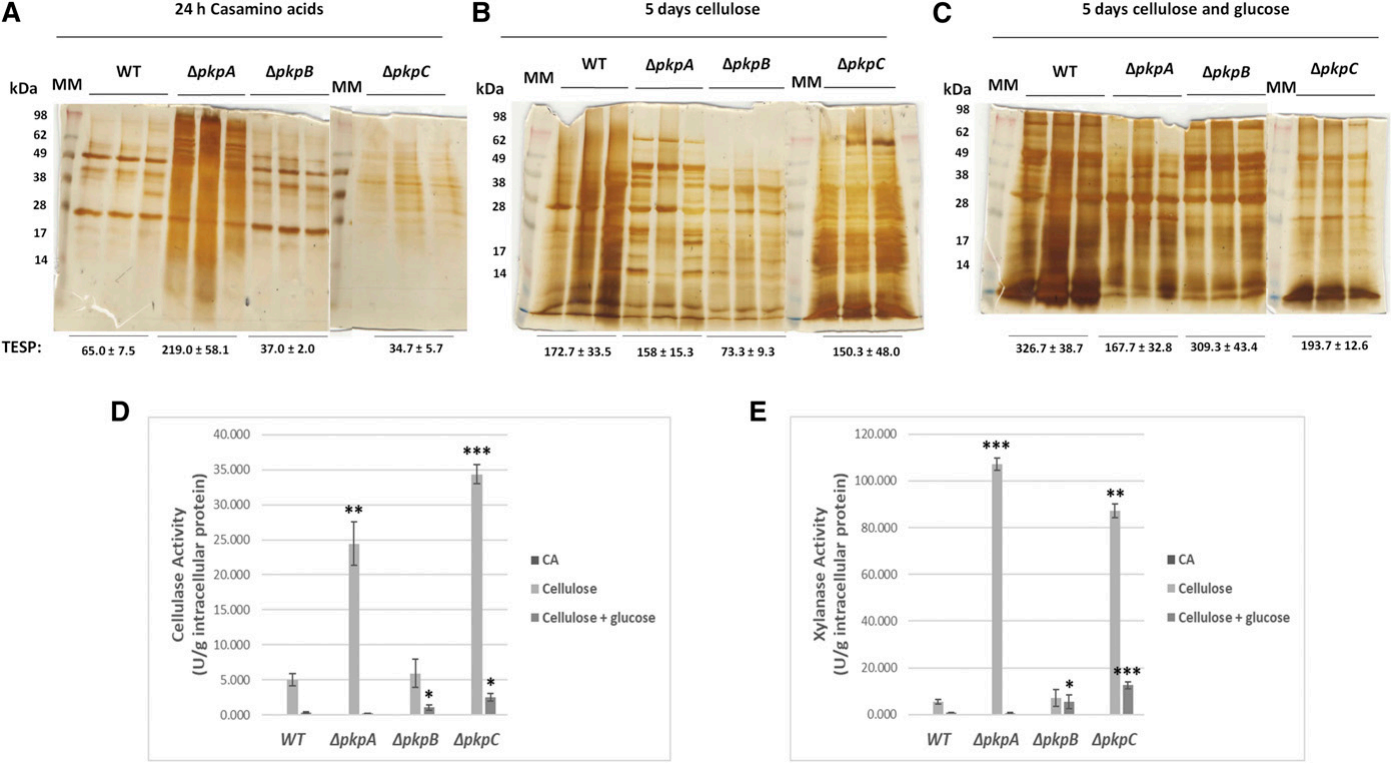
PkpA and PkpC are involved in hydrolytic enzyme secretion. A., B., C. Silver-stained protein gels showing biological triplicates of 20 μl total protein from 10 × concentrated culture supernatants of the wild-type and the pyruvate dehydrogenase protein kinase (PDHK) deletion strains when grown for 24 h in minimal medium supplemented with casamino acids and after transfer to cellulose-rich or cellulose- and glucose-rich medium for 5 days. Total extracellular secreted protein (TESP) concentrations, as determined by Bradford assay, for each biological triplicate are also shown. D., E. Cellulase and xylanase activities in the supernatants of the wild-type and PDHK deletion strains when grown in the same above specified conditions. Enzyme activities were normalized by intracellular protein concentration. Standard deviations present the average of 3 biological replicates (*P-value < 0.05; **P-value < 0.005; ***P-value < 0.0005 as determined by a one-tailed, paired student *t*-test).

One of the aims of this work was to assess the involvement of the PDHKs in enzyme secretion, such as cellulases and xylanases, required for lignocellulosic biomass deconstruction in the presence of biotechnologically-relevant carbon sources. Total protein secretion as well as cellulase and xylanase activities were therefore assessed in culture supernatants when the wild-type and the PDHK deletion strains were grown for 24 h in casamino acid-rich medium and then transferred to minimal medium supplemented with cellulose or cellulose and glucose for 5 days (glucose was supplied every 48 h to a final concentration of 1% v/v in order to assure continuous CCR). Deletion of *pkpA* and *pkpC* resulted in a total protein secretion profile that differed substantially from the wild-type strain in the presence of casamino acids, cellulose and cellulose and glucose (Figure 3A-C), suggesting that both PDHKs are involved in protein secretion. In agreement, extracellular cellulase (24.4 ± 3.1 and 34.3 ± 1.34 U/g intracellular protein) and xylanase (107.3 ± 2.6 and 87.1 ± 2.1 U/g intracellular protein) activities were significantly [P-values: (cellulase activity 0.0008 and 0.0001); (xylanase activity 4.02 × 10^−6^ and 0.0007)] increased in the Δ*pkpA* and Δ*pkpC* strains when compared to the wild-type strain (5.04 ± 0.89 and 5.6 ± 1.0 U/g intracellular protein) when grown for 5 days in cellulose-rich medium (Figure 3D, E). The continuous supply of glucose to the cellulose-grown cultures resulted in cellulase and xylanase activities repression in all strains (Figure 3D, E), indicating that CCR is functional. However, several significant differences in cellulase and xylanase activities were observed between the strains. In the simultaneous presence of cellulose and glucose, the Δ*pkpC* strain presented significant increased cellulase (2.5 ± 0.6 U/g intracellular protein) and xylanase (12.5 ± 1.6 U/g intracellular protein) activity when compared to the wild-type strain (0.33 ± 0.15 and 0.93 ± 0.13; P-values 0.008 and 0.00011 respectively) and which was statistically insignificant to the activities observed for the wild-type strain (5.04 ± 0.89 and 5.6 ± 1.0 U/g intracellular protein) when grown solely in cellulose-rich medium (Figure 3D, E). The Δ*pkpB* strain presented no significant statistical difference in extracellular cellulase (P-value: 0.27) and xylanase (P-value: 0.27) activities in the presence of cellulose; whereas in the simultaneous presence of glucose and cellulose this strain also had significantly (P-values of 0.04 and 0.03) increased hydrolytic enzyme activities (1.1 ± 0.37 and 5.4 ± 2.8) when compared to the wild-type strain (0.33 ± 0.15 and 0.93 ± 0.13). These results indicate a defect in glucose metabolism and/or CCR in the Δ*pkpB* and Δ*pkpC* strains, resulting in an increase in hydrolytic enzyme secretion in these strains in the presence of glucose. Furthermore, the total extracellular secreted protein (TESP), as measured by Bradford assay, was significantly lower in the presence of glucose and cellulose, and slightly lower in the presence of cellulose in the Δ*pkpC* strain when compared to the wild-type strain, indicating that PkpC was involved in cellulase and xylanase secretion and not in total protein secretion (Figure 3). The same was also observed for the Δ*pkpA* strain in the presence of cellulose and cellulose and glucose (Figure 3). TESP was also lower in the Δ*pkpB* strain in the presence of casamino acids and cellulose but not in the simultaneous presence of glucose and cellulose, where an increase in cellulase and xylanase activity was observed, suggesting that PkpB is not specifically involved in the secretion of these enzymes but may be involved in the secretion of additional (non)-hydrolytic enzymes (Figure 3). In summary, these results show that PkpA and PkpC, are important for cellulase and xylanase secretion in the presence of the biotechnologically-relevant carbon sources.

### pkpA and pkpC genetically interact in the presence of glucose and xylose

**Figure 4:**
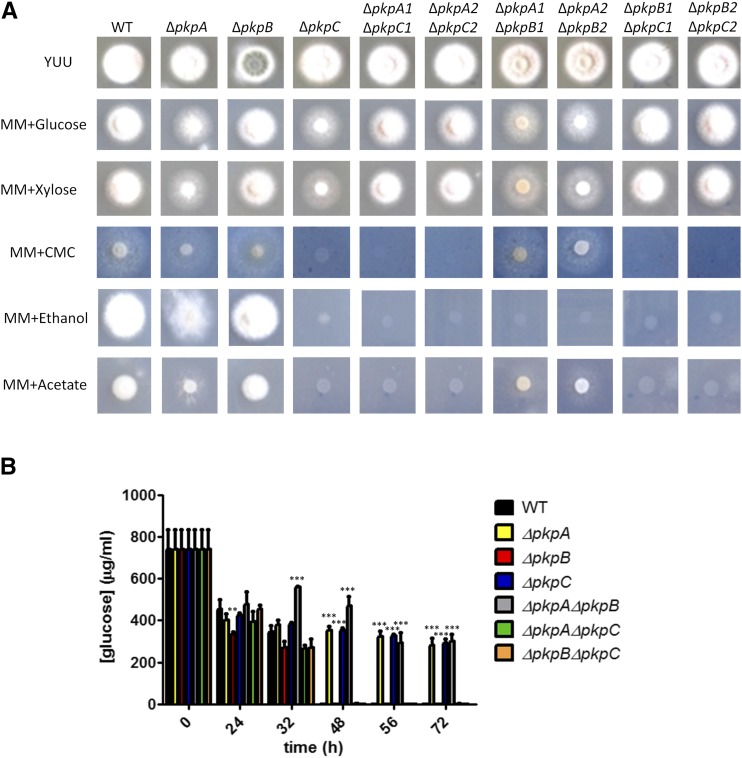
Genetic interaction between the pyruvate dehydrogenase protein kinase-encoding genes. A. Growth of the wild-type (TN02a3), Δ*pkpA*, Δ*pkpB*, Δ*pkpC* and the double deletion strains in the presence of 1% w/v of different carbon sources. B. Glucose consumption, measured indirectly by assessing the concentration of extracellular glucose, of the WT single and double deletion strains when grown directly in minimal medium supplemented with glucose for 72 h. Standard deviations represent the average of 3 biological replicates (**P-value < 0.01; ***P-value < 0.001 as determined by a two-way ANOVA test and Bonferroni post-tests).

To study a potential genetic interaction between the three PDHKs, double mutants were generated by crossing the single gene deletion strains with each other and the presence of both deletion mutations was confirmed by PCR. Two independent segregants for each homologous integrated double deletion strain were chosen for further analysis. The wild-type, single and double deletion strains were then once more grown in the presence of different carbon sources and compared to the respective parental strains (Figure 4A): i) the Δ*pkpA* Δ*pkpB* strain had the same growth phenotype as the Δ*pkpA* strain in all tested carbon sources; ii) the Δ*pkpA* Δ*pkpC* presented growth similar to the Δ*pkpC* strain in the presence of the alternative carbon sources acetate, ethanol and CMC, but had a wild-type growth phenotype in the presence of glucose and xylose; iii) the Δ*pkpB* Δ*pkpC* strain grew like the Δ*pkpB* strain in the presence of glucose and xylose and similar to the Δ*pkpC* strain in the presence of the alternative carbon sources. These results indicate that *pkpA* and *pkpC* genetically interact in the presence of glucose and xylose as growth did not resemble any of the parental strains in these carbon sources; whereas interaction between *pkpB* and *pkpC* appears to be carbon source-dependent. On the other hand, *pkpA* and *pkpB* do not appear to genetically interact.

To further characterize the double deletion mutants, they were grown in the presence of increasing concentrations of allyl alcohol (AA) in glucose-rich medium. The Δ*pkpA* Δ*pkpB* and Δ*pkpB* Δ*pkpC* strains presented the same resistance to 20 mM AA than the wild-type or Δ*pkpB* strains, whereas growth was reduced in the presence of 40 mM AA, although not to the same levels than the Δ*pkpA* and Δ*pkpC* single deletion strains (Figure 1B-C). The Δ*pkpA* Δ*pkpC* strain presented increased sensitivity to AA, although AA sensitivity was not as severe as for the parental single deletion strains (Figure 1B-C). These results further support a genetic interaction between *pkpA* and *pkpC* and also suggest a genetic interaction between *pkpB* and *pkpA* or *pkpC* in the presence of increased concentrations of AA.

Next, glucose uptake was measured in the double deletion strains when grown directly in minimal medium supplemented with glucose for 72 h and compared to the wild-type and respective parental strains (Figure 4C). All double deletion strains, with the exception of the Δ*pkpA* Δ*pkpB* strain, consumed all extracellular glucose within 48 h (Figure 4C). The Δ*pkpA* Δ*pkpC* and Δ*pkpB* Δ*pkpC* strains therefore presented a glucose consumption profile similar to the wild-type strain and distinct from their parental strains (*e.g.*, the Δ*pkpA* and Δ*pkpC* parental strains have a severely reduced glucose consumption profile). The Δ*pkpA* Δ*pkpB* strain on the other hand, had the same reduced glucose consumption profile as the Δ*pkpA* single deletion strain (Figure 4C).

In summary, these results suggest that *pkpA* and *pkpC* genetically interact in the presence of glucose and xylose with the deletion of both genes resulting in growth phenotypes and a glucose consumption profile similar to the wild-type strain. The Δ*pkpB* Δ*pkpC* strain presented growth phenotypes that were similar to the Δ*pkpC* single deletion strain in the presence of alternative carbon sources whereas growth on glucose, glucose consumption and sensitivity to AA was similar to the Δ*pkpB* single deletion strain or presented an intermediate phenotype, suggesting condition-dependent genetic interaction. Glucose consumption and growth profiles suggest that no interaction appears to occur between *pkpA* and *pkpB*, whereas a slight increase in AA sensitivity was observed, also suggesting a condition-dependent genetic interaction.

### PkpA and PkpC are important for glucose-related growth and consumption that affected carbon catabolite repression (CCR)

The above described results indicate defects in the utilization of glucose. To further investigate the roles played by PkpA and PkpC during growth on glucose, microscopy was carried out when the wild-type, Δ*pkpA-C* strains were grown for 7 h at 37° or 16 h at 30° in minimal medium supplemented with 1% w/v glucose. In agreement with the observed reduction of growth on solid, glucose-rich media after 48 h, deletion of *pkpA* and *pkpC* also caused a statistically significant reduction in germ tube emergence (∼50%) and subsequent delay in hyphal growth when compared to the wild-type and Δ*pkpB* strains in the presence of glucose after 7 h or 16 h (Figure S2C). The observed glucose-related growth defect was abolished after prolonged incubation, as radial diameters of the Δ*pkpA* and Δ*pkpC* when grown for 5 days on minimal medium supplemented with glucose was equal to the wild-type strain (Figure 1B).

**Figure 5:**
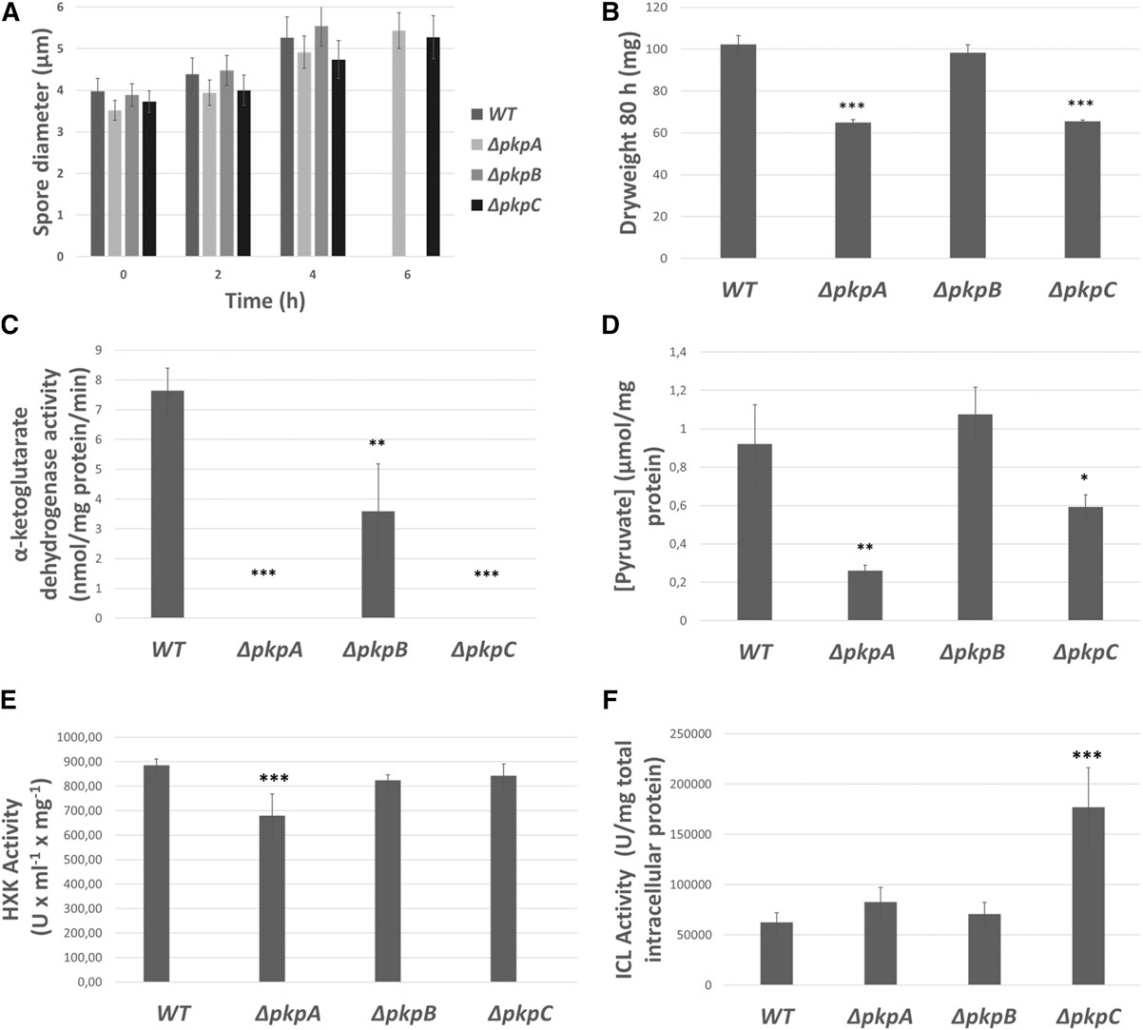
PkpA and PkpC are important for glucose uptake, carbon catabolite repression and metabolism. A. Glucose sensing is intact in the *pkp* deletion mutants. Spore diameter of the wild-type and protein kinase deletion strains after 0 h (control), 2 h, 4 h and 6 h incubation in minimal medium supplemented with 1% w/v glucose at 37°C, 160 rpm. Standard deviations present the average size of 100 spores of three biological replicates. B. Dryweight of the strains grown in the above specified conditions is also shown. C. alpha-ketoglutarate dehydrogenase activity and D. intracellular pyruvate levels. All strains were grown for 24 h in casamino acid-rich medium and then transferred to minimal medium supplemented with glucose for 1 h. E. Hexokinase (HXK) and F. isocitrate lyase (ICL) activities in the wild-type and *pkp* deletion mutants when grown in the same conditions as specified under C. and D. with the exception that strains were incubated for 6 h in these conditions. Standard deviations represent the average of 3 biological replicates (*P-value < 0.05; **P-value < 0.005; ***P-value < 0.0005 as determined by a one-tailed, paired student *t*-test).

Initial glucose-related growth difficulties may be due to either a defect in glucose sensing, consumption and/or metabolism. Fungal germination can be distinguished into two main separate events: i) conidia swelling which is triggered when the fungus senses a suitable extracellular carbon source such as D-glucose and ii) germ tube emergence and hyphal outgrowth where the respective sugar is actively taken up and metabolized to sustain hyphal growth (Hayer *et al.* 2013). To investigate the first hypothesis, conidia swelling of the wild-type and the three protein kinase deletion strains were assessed over a time period of 6 h in the presence of glucose (Figure 5A). All four strains presented conidia swelling over the first 4 h, but whereas a germ tube had emerged in the wild-type and Δ*pkpB* strains after 6 h in most conidia, mainly large swollen conidia without a germ tube were observed for the Δ*pkpA* and Δ*pkpC* strains (Figure 5A and Figure S3). These results suggest that the Δ*pkpA* and Δ*pkpC* strains are able to sense glucose properly, but that germ tube emergence and subsequent hyphal elongation is delayed.

Next, glucose consumption was assessed in the wild-type and the protein kinase deletion strains when grown directly from the same amount of conidia in minimal medium supplemented with glucose for a period of 72 h. The wild-type and Δ*pkpB* strains consumed all extracellular glucose within 48 h whereas the Δ*pkpA* and Δ*pkpC* strains still had not taken up all available glucose after 72 h (Figure 4C). In agreement, a significant reduction in fungal biomass was observed after 72 h for the Δ*pkpA* and Δ*pkpC* strains when compared to the wild-type strain (Figure 5B). These results confirm a delay/reduction in glucose consumption in the Δ*pkpA* and Δ*pkpC* strains, which was not due to a particular lack of glucose transporter gene expression (despite substantial fluctuations in gene expression in all tested strains), as shown by RT-qPCR of the hexose transporter-encoding genes *hxtB-E* when strains were incubated in minimal medium supplemented with glucose for different time periods (Figure S4).

Glucose uptake and subsequent phosphorylation during the first step of glycolysis by hexokinase (HXK) has previously been shown to act as a signal for the carbon catabolite repressor CreA::GFP to translocate to the nucleus where it represses genes required for the use of alternative carbon sources (Brown *et al.* 2013). Consistent with the above described defect in glucose consumption, CreA::GFP did not translocate to the nuclei in the Δ*pkpA* (0%) and Δ*pkpC* (0%) strains (Figure S5A) whereas it did so in the wild-type (77.4%) and Δ*pkpB* (77.5%) strains when grown for 16 h in glucose-rich medium. Furthermore, protease secretion, induced during growth on dry-skimmed milk and repressed in the presence of glucose, was not inhibited in the Δ*pkpA* and Δ*pkpC* strains (Figure S5B). These observations suggest that the reduced glucose consumption profile resulted in delayed germination and growth, as well as in the absence of the signal that is required for CCR.

### PkpA and PkpC are important for glucose metabolism

The above described defects in glucose utilization may also result from defects in glucose metabolism. To determine whether the deletion of *pkpC* resulted in defects in glycolytic enzyme activities, interactions of PkpC::GFP with other proteins, when grown for 24 h in casamino acids and then transferred to glucose-rich medium for 10, 30 and 60 min, were determined (File S1). After 10 min incubation in glucose-rich medium, a putative interaction of PkpC with the ATP citrate synthase AclB was observed (File S1). AclB is a TCA (tricarboxylic acid) cycle enzyme, converting Acetyl-CoA and oxaloacetate to citrate, therefore catalyzing the first step in the Krebs cycle and allowing the TCA cycle to occur in the mitochondria (Hynes and Murray 2010). It is possible that PkpC regulates the activity of AclB upon exposure to high concentrations of glucose whereas deletion of *pkpC* may result in aberrant TCA cycle progression. Indeed, the activity of the TCA cycle enzyme α-ketoglutarate dehydrogenase was close to zero in the Δ*pkpA* and Δ*pkpC* strains after 1 h incubation in the presence of glucose (Figure 5C), suggesting that both protein kinases are required for TCA cycle progression. Furthermore, intracellular pyruvate levels were also significantly decreased in these two deletion strains in the same conditions (Figure 5D). A decrease in intracellular pyruvate concentration may result from either reduced glucose metabolism or increased pyruvate metabolism, namely through the action of the PDH. The activity of HXK, was subsequently assessed when strains were grown for 24 h in minimal medium supplemented with casamino acids and after transfer to glucose-rich medium for 1 h. HXK activity was similar between the wild-type, Δ*pkpB* and Δ*pkpC* strains, whereas it was significantly reduced in the Δ*pkpA* strain when compared to the wild-type strain (Figure 5E). These results indicate that PkpA is important for the metabolic utilization of glucose. An overview of the observed phenotypes and regulation of metabolic enzymes in the presence of glucose is given in Figure 8A.

### Metabolome analysis of the wild-type, ΔpkpA, ΔpkpB and ΔpkpC strains in the presence of glucose and cellulose

To further assess the effect of the deletion of *pkpA-C* on cellular metabolism, metabolome analysis of the wild-type and the respective deletion strains was performed in the presence of glucose and cellulose. All strains were grown for 24 h in casamino acid-rich medium (to get enough biomass) before being transferred to minimal medium supplemented with either glucose or carboxymethylcellulose (CMC) for 16 h and 48 h respectively. The 16 h time point for glucose was chosen as the fungus is still metabolising glucose and has not entered starvation yet. The 48 h time point for CMC allowed induction of cellulase- and hemicellulase-encoding genes and the start of secretion of enzymes required for cellulose breakdown (data not shown). The levels of the respective identified metabolites were compared between four biological replicates of the PDHK deletion and wild-type strains in both conditions and raw data were normalized by fungal dry weight (Files S2-S3).

**Figure 6:**
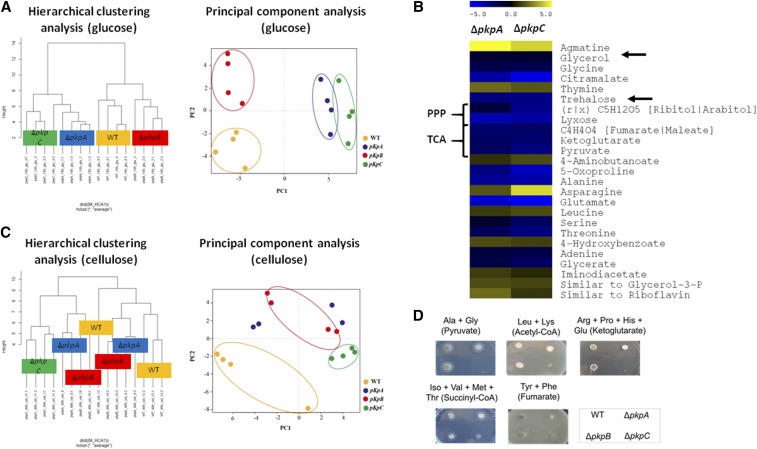
Primary metabolism is significantly altered in the *pkpA* and *pkpC* deletion mutants in the presence of glucose and cellulose. A., C. Hierarchical clustering and principal component analysis of the levels of metabolites identified in the wild-type (WT) and pyruvate dehydrogenase kinase (*pkp*) deletion strains when incubated for 16 h and 48 h in minimal medium supplemented with glucose or cellulose respectively. B. Heat map showing the Log2-fold change of metabolite levels that were significantly different between the WT and Δ*pkpA* and Δ*pkpC* strains after 16 h incubation in glucose-rich conditions. Arrows indicate the intracellular storage compounds glycerol and trehalose whereas pentose phosphate pathway (PPP) and TCA (tricarboxylic acid) cycle intermediates are also indicated. D. Growth of the wild-type and *pkp* deletion mutants in the presence of TCA cycle precursors. Medium was supplemented with 50 mM of each amino acids and plates were inoculated with 10^5^ spores.

In the presence of glucose, the Δ*pkpA*, Δ*pkpB* and Δ*pkpC* strains clustered [as determined by hierarchical clustering analysis (HCA) and principal component analysis (PCA)], apart from the wild-type strain (Figure 6A). Whereas the wild-type and Δ*pkpB* strains clustered closer together, the Δ*pkpA* and Δ*pkpC* strains clustered wider apart from these two strains (Figure 6A). These results support the above described observations (Figure 5B-C) that glucose consumption and metabolism is disturbed in these strains. Levels of the intracellular storage compounds trehalose (Log2 of -2.5 and -2.6) and glycerol (Log2 of -0.93 and -0.9) were significantly reduced in the Δ*pkpA* and Δ*pkpC* strains and increased in the Δ*pkpB* strain (Log2 of 1.4 and 1.1) when compared to the wild-type strain (Figure 6B). Trehalose is a reserve carbohydrate which provides energy for the cell during conidia germination and hyphal development (Thevelein 1984). In addition, trehalose also protects the fungal cell from environmental stresses and nutrient deprivation (Al-Bader *et al.* 2010). Similarly, glycerol also serves as an intracellular carbohydrate reserve and protects against osmotic stress (Hondmann *et al.* 1991). In agreement with the above described defects in glucose uptake and metabolism, this data suggests that the Δ*pkpA* and Δ*pkpC* strains are undergoing carbon starvation in glucose-rich conditions.

**Figure 7:**
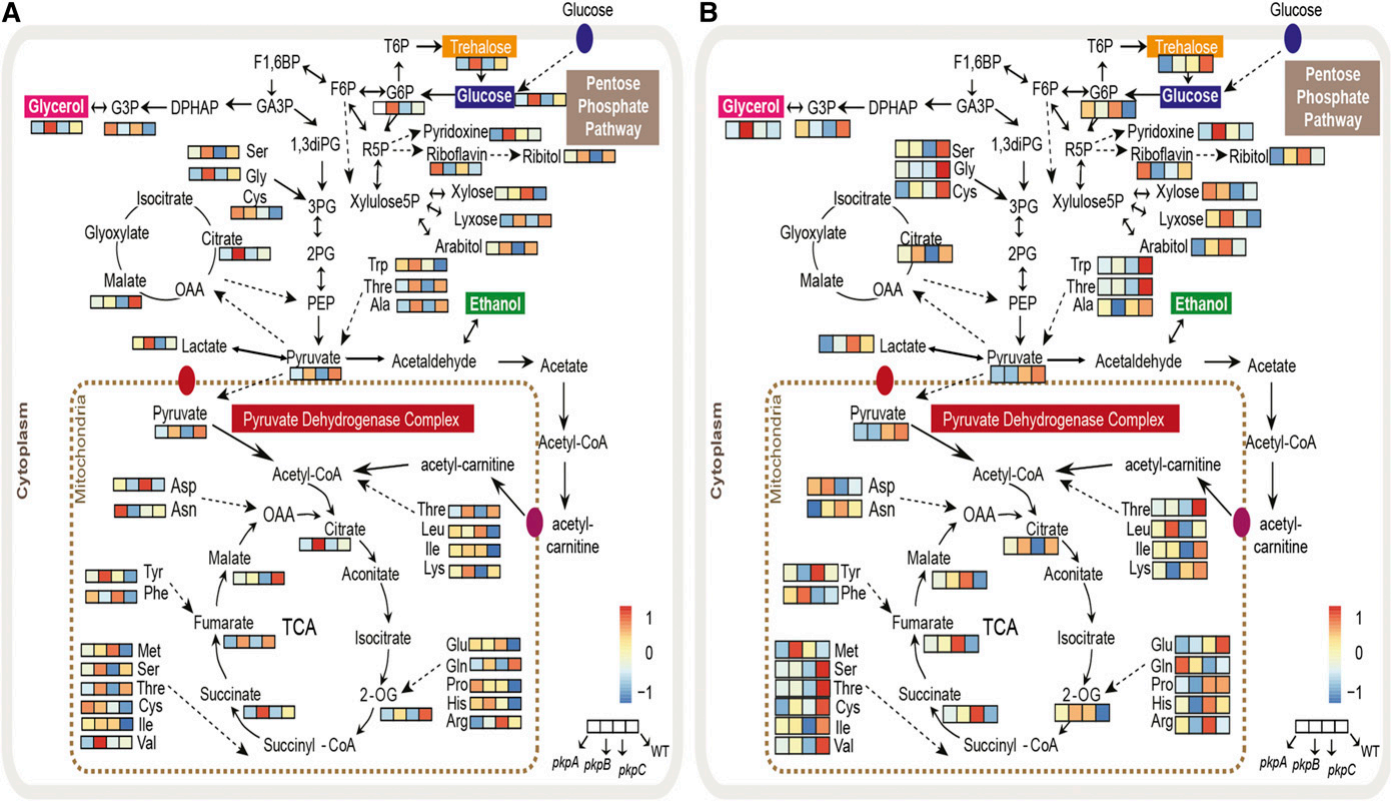
Levels of identified metabolites and the pathways in which they are produced in the wild-type and pyruvate dehydrogenase kinase deletion strains. A., B. Maps of primary metabolism (glycolysis, TCA cycle and pentose phosphate pathway) showing the average levels of identified pathway-specific metabolites of 4 biological replicates in the wild-type (WT), Δ*pkpA*, Δ*pkpB* and Δ*pkpC* strains when grown for A. 16 h in glucose-rich or for B. 48 h in cellulose-rich medium.

Furthermore, analysis of the levels of the metabolic intermediates of the pentose phosphate pathway and the TCA cycle, showed a significant decrease in the Δ*pkpA* and Δ*pkpC* strains when compared to the wild-type strain, suggesting a down-regulation of these pathways (Figure 6B). This is in agreement with the above described reduction in α-ketoglutarate dehydrogenase (KGDH) activity in the presence of glucose. Moreover, levels of several amino acids were also decreased in both protein kinase deletion strains when compared to the wild-type strain, suggesting the use of amino acids, such as alanine and glutamate, as carbon sources, that can by readily converted to pyruvate and α-ketoglutarate respectively (Figure 6B). An overview of the levels of the identified metabolites and the specific pathway in which they are produced are shown in Figure 7A.

In the presence of cellulose, the Δ*pkpC* strain, as determined by HCA and PCA, clearly clustered apart from the other three strains (Figure 6C), supporting the above described cellulose-related growth defects of the Δ*pkpC* strain. Furthermore, several TCA cycle intermediates were either increased or decreased in the Δ*pkpC* strain but not in the other three strains, suggesting a defect in the TCA cycle in this strain (File S3). Indeed, the Δ*pkpC* strain grew significantly less on TCA cycle intermediate precursors, especially those feeding into the first half of the cycle, when compared to the wild-type and the other two PDHK deletion strains (Figure 6D), suggesting a de-regulation in central carbon metabolism. An overview of the levels of the identified metabolites and the pathways in which they are produced is given in Figure 7B.

### PkpC is predicted to regulate enzymes required for acetate metabolic utilization

The Δ*pkpA* and Δ*pkpC* strains presented strong growth defects when grown solely in the presence of acetate with the deletion of *pkpC* completely inhibiting growth in this condition (Figure 1A). To determine the role played by PkpC in acetate metabolism, protein-protein interactions of the GFP tagged PkpC protein in the presence of acetate were determined. PkpC::GFP was grown for 24 h in casamino acid-rich medium before being transferred to minimal medium supplemented with acetate for 10, 30 and 60 min. Identified proteins from both conditions (casamino acids and acetate) were classified according to MIPS (Munich Information Centre for Protein Sequences) (File S4). After 60 min incubation in acetate, putative interactions between PkpC and nine other protein kinases (of which four are essential), including the 6-phosphofructokinase PfkA and the TOR (Target of Rapamycin) kinase TorA, were observed (File S4). PfkA plays a role in gluconeogenesis and glycolysis through catalyzing the phosphorylation of fructose-6-phosphate to fructose-1,6-biphosphate, in the third step of glycolysis. TorA is the homolog of the two *S. cerevisiae* TOR protein kinases Tor1p/Tor2p, which form two distinct complexes and govern a myriad of different cellular processes such as nutrient sensing, translation and ribosome biogenesis (Rohde *et al.* 2008). In addition, PkpC was shown to potentially interact with two other TOR-associated proteins in the presence of acetate, including the ortholog of *S. cerevisiae* Kog1p (TOR1 complex subunit) and Lst8p (associates with the TOR1/2 complex during nitrogen uptake) (File S4). In *S. cerevisiae*, ribosome biogenesis, including the transcription of genes encoding 35S and 5S rRNAs and tRNAs, are targeted by Tor signaling (Rohde *et al.* 2008). Potential interactions between PkpC and various proteins of RNA metabolic processes, such as spliceosome components or ribosomal unit assembly were observed (File S4), reinforcing a link with TOR-mediated signaling.

Moreover, putative interactions of PkpC with enzymes of different metabolic pathways, such as gluconic, amino and fatty acid, were observed (File S4). After 60 min incubation in acetate-rich media, potential interactions with a TCA cycle succinate CoA-ligase, the pyruvate decarboxylase PdcA and several enzymes involved in two-carbon compound metabolism were also observed (Table 1). The latter included the acetate permease AcpA, the malate synthase AcuE, the alcohol dehydrogenase (ADH) AlcA and the isocitrate lyase (ICL) AcuD (Table 1). To confirm these data, ICL activity was measured in the wild-type and PDHK deletion strains when grown in acetate-rich medium and found to be increased around threefold in the Δ*pkpC* strain (Figure 5F), suggesting that PkpC negatively regulates ICL activity. These results indicate a central role for PkpC in the regulation of enzymes required for acetate transport and metabolism. It is therefore probable that the absence of *pkpC* is responsible for a mis-regulation of these enzymes, causing an inability to use acetate as the sole carbon and energy source. Furthermore, the lack of potential interactions with other protein kinases, including the nutrient sensor TorA, and regulation of RNA metabolic processes may also have detrimental effects for the cell in the Δ*pkpC* strain.

**Table 1:** Proteins identified by mass-spectrometry which putatively interact with PkpC after 60 min incubation in acetate-rich media

Gene ID	Description	Type of metabolism
AN1895	MaiA; maleyl-acetoacetate isomerase (phenylalanine catabolism)	Amino acid
AN1883	Putative argininosuccinate synthase (arginine metabolism)	Amino acid
AN5701	AroF; putative 3-deoxy-D-arabino-heptulosonate 7-phosphate synthase (aromatic amino acid biosynthesis)	Amino acid
AN5604	AcuG; putative fructose-bisphosphatase	Central (gluconeogenesis/glycolysis)
AN3649	Uncharacterized ORF; orthologs have mitochondrial ribosome localization	Fatty acid
AN1409	Putative acetyl-CoA C-acetyltransferase	Fatty acid
AN10834	AcdB; protein with an acyl-CoA dehydrogenase domain	Fatty acid
AN4888	PdcA; putative pyruvate decarboxylase	Pyruvate
AN8979	AlcA; alcohol dehydrogenase	Two-carbon compound
AN5226	AcpA; acetate permease (acetate uptake)	Two-carbon compound
AN6653	AcuE; malate synthase, required for utilization of acetate as carbon source	Two-carbon compound
AN5669	Putative succinyl-CoA:3-ketoacid-coenzyme A transferase	Two-carbon compound
AN7632	Putative dehydrogenase	Two-carbon compound
AN5634	AcuD; isocitrate lyase, required for utilization of acetate and fatty acids as carbon sources	Two-carbon compound/Fatty acid

## Discussion

The conversion of pyruvate to acetyl-coA by the mitochondrial pyruvate dehydrogenase complex (PDH) presents a core enzymatic reaction in carbon metabolism, linking glycolysis to the TCA cycle, which ultimately leads to ATP production (Kolobova *et al.* 2001). The activity of the PDH is controlled by a combination of PDHKs and PDHPs which have been shown to be important for development, pathogenicity and enzyme secretion in different fungal species (de Assis *et al.* 2015a; Gao *et al.* 2016; Steensma *et al.* 2008; Brown *et al.* 2013). *Aspergillus* consists of several fungal species that have various biotechnological applications or are of medical importance. This work therefore set out to characterize the role of three PDHKs in the utilization of the biotechnologically important carbon sources glucose and cellulose and the physiologically relevant carbon source acetate in the *Aspergillus* reference organism *A. nidulans*, in order to uncover additional carbon utilization routes which may be of importance for these applications.

### Evolutionary and functional divergence of PkpA, PkpB and PkpC

Phylogenetic analysis of the three predicted *A. nidulans* PDHKs and the respective homologs in other filamentous and yeast-like fungi, showed that PkpC and homologs clustered apart from the other two PDHKs. Of the three *A. nidulans* PDHKs, only PkpC was identified as the true ortholog of the four human PDHK isozymes (OrthoMCL database) (Chen *et al.* 2006), indicating an evolutionary functional divergence or origin of this enzyme. This corroborates the here presented results, where deletion of *pkpC*, but not *pkpA* or *pkpB* resulted in a severe growth defect in the presence of a wide range of carbon sources. A detailed phylogenetic analysis of PDHK-encoding genes in other fungi, including *S. cerevisiae* has not been carried out to date, but deletion of the respective genes also resulted in growth defects (Gao *et al.* 2016; Steensma *et al.* 2008) and in *S. cerevisiae*, distinct functions for both PDHKs were also observed (Steensma *et al.* 2008), indicating a functional divergence in fungal PDHKs.

### PkpA, but not PkpC, regulates PDH activity

Despite PkpC being predicted to be the true ortholog of human PDHKs, cellular localization of the GFP-tagged strain and activity of PDH in the *pkpC* deletion strain, do not support this prediction. PkpA and PkpB were localized to the mitochondria in both preferred and alternative carbon sources, whereas mitochondrial localization of PkpC occurred only in the presence of acetate and not in glucose-rich conditions. PkpC is not responsible for regulating PDH activity (indeed PkpC is cytoplasmic in glucose-rich conditions) and protein interaction studies showed no interaction of PkpC with any of the PDH subunits in glucose-rich conditions. In *S. cerevisiae*, the two PDHKS Pkp1p and Pkp2p were shown to be predominantly mitochondrial and to regulate PDH activity (Gey *et al.* 2008; Krause-Buchholz *et al.* 2006).

PkpA was shown to positively regulate PDH activity in the presence of glucose, as observed by a reduction in PDH activity in the Δ*pkpA* strain. PkpA may regulate PDH activity directly or indirectly, through interaction with an additional protein that is important for PDH activity. In contrast to *A. nidulans*, deletion of the *S. cerevisiae PKP2*, but not *PKP1*, resulted in increased PDH activity (Gey *et al.* 2008). Positive regulation of the PDH is thought to occur by de-phosphorylation, catalyzed by protein phosphatases (Patel and Korotchkina 2006; Gey *et al.* 2008; Krause-Buchholz *et al.* 2006; Wieland *et al.* 1972). In other fungi (Gao *et al.* 2016; Steensma *et al.* 2008), including *S. cerevisiae*, where Pkp1p and Pkp2p phosphorylate the α-subunit of PDH (Gey *et al.* 2008; Krause-Buchholz *et al.* 2006), phosphorylation of PDH was shown to negatively regulate the activity of the PDH, which is in agreement with the predicted function for these type of enzymes (Patel and Korotchkina 2006; Gey *et al.* 2008). These results therefore suggest a novel function for a protein kinase governing the activity of PDH in *A. nidulans*. The exact roles (if any) of PkpA and PkpB in regulating PDH activity, as well as assessing the phosphorylation state of each of the PDH subunits in the *pkpA* and *pkpB* deletion mutants, is subject to further investigation.

### PkpA and PkpC are involved in regulating glucose utilization

The Δ*pkpA* and Δ*pkpC* strains presented several defects in glucose utilization, that were found to be non-sensing-related. Glucose consumption was delayed and shown to be independent of glucose transporter gene expression-dependent and of putative interactions between PkpC and any glucose transporters. It is possible that the interaction between PkpC and a putative glucose transporter may be weak and/or transient and could have been missed during mass spectrometry in the here defined conditions.

**Figure 8:**
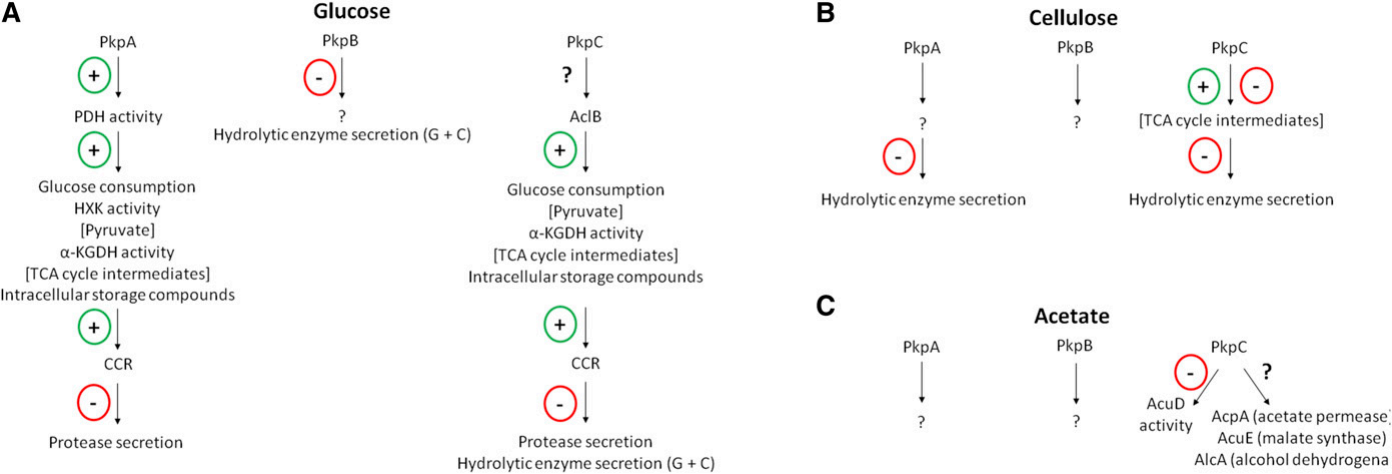
Schematic overview of enzymes and cellular processes regulated by PkpA, PkpB and PkpC when growing on different carbon sources. A. In the presence of glucose, PkpA positively regulates the activity of the PDH (pyruvate dehydrogenase complex) either directly or indirectly, allowing glucose consumption, glycolysis (HXK – hexokinase), the TCA (tricarboxylic acid) cycle (α-KGDH – α-ketoglutarate dehydrogenase) and carbon catabolite repression (CCR) to occur. In the absence of this protein kinase, the aforementioned metabolic pathways are reduced, resulting in faulty CCR and increased protease secretion. The targets of PkpB are currently unknown, but deletion of the corresponding gene resulted in increased hydrolytic enzyme secretion in the simultaneous presence of glucose and cellulose (G + C). PkpC is predicted to regulate the activity of the ATP citrate synthase AclB, therefore indirectly ensuring glucose consumption, glycolysis and TCA cycle progression, and CCR. In the absence of *pkpC*, the aforementioned metabolic pathways are reduced, resulting in faulty CCR and increased protease and hydrolytic enzyme secretion in the presence of G + C. Additional protein targets are likely to exist that were not detected in the here defined conditions. B. In the presence of cellulose, the specific targets of PkpA, PkpB and PkpC remain undefined. Deletion of *pkpA* and *pkpC* resulted in increased hydrolytic enzyme secretion in this carbon source, with the latter strain presenting different concentrations of TCA cycle intermediates. C. In the presence of acetate, PkpA and PkpB protein targets remain unkown, whereas PkpC negatively regulates the activity of the glyoxylate cycle enzyme, isocitrate lyase (ICL) AcuD. Furthermore, putative interactions of PkpC with additional glyoxylate cycle enzymes and an acetate permease are predicted; this protein kinase therefore being crucial for acetate metabolism.

In addition, the reduction in glucose consumption may also be a direct result of a mis-regulation in primary glucose metabolism. PkpC was shown to potentially interact with AclB, a subunit of the ATP citrate synthase, an enzyme that ensures TCA cycle progression. The specific role of PkpC in AclB regulation remains to be determined, but a hyper- or in-activation of this enzyme would be detrimental for TCA cycle progression. Indeed, a complete inhibition of α-ketoglutarate dehydrogenase activity and reduction in TCA cycle intermediates in the presence of glucose was observed, supporting a block in glucose metabolism, which would impair glucose uptake and subsequent carbon catabolite repression (CCR), as shown by the absence of CreA in the nucleus in these strains (Figure 8A). Deletion of *aclB* in *A. nidulans*, resulted in severely reduced growth in the presence of glucose which was predicted to be due to defects in acetyl-coA metabolism (Hynes and Murray 2010). A similar situation has previously been described in *A. nidulans*, where the deletion of several phosphatase-encoding genes resulted in, in addition to an inability to transport glucose due to reduced glucose transporter gene expression, reduced α-ketoglutarate dehydrogenase (KGDH) activity, thereby blocking TCA cycle progression and abolishing growth in the presence of glucose (de Assis *et al.* 2015a). The same study also identified the PDHPs PtcD and PtcE as being required for growth on glucose, including consumption, respiration and metabolism (de Assis *et al.* 2015a).

A similar situation can be envisaged for the *pkpA* deletion strain although the mechanism is likely to be different from the Δ*pkpC* strain (Figure 8A). A decrease in PDH activity in the Δ*pkpA* strain results in reduced glucose metabolic flow, as supported by a reduction in the activity of hexokinase (HXK) and in severely reduced intracellular pyruvate levels (Figure 8A). This disturbance in glycolysis subsequently de-regulates the TCA cycle, as shown by reduced levels of TCA cycle intermediates and inhibition of α-ketoglutarate dehydrogenase activity, further impacting glucose consumption and CCR (Figure 8A). In *S. cerevisiae*, disruption of either *PKP1* or *PKP2* had no significant effect on growth on glucose (Steensma *et al.* 2008), suggesting a fungal-specific divergence in function of these type of enzymes.

The aforementioned metabolic disturbances force the Δ*pkpA* and Δ*pkpC* strains to consume the intracellular storage compounds trehalose and glycerol (8A). Although glucose metabolism was disturbed in these strains, these impairments seem to be especially strong during the first 24 h – 48 h incubation in glucose-rich conditions. Prolonged incubation times, resulted in increased glucose consumption and restored growth of both strains on solid glucose-rich medium.

Although deletion of *pkpB* had a less severe impact on glucose metabolism than the deletion of the other two PDHK-encoding genes, this strain nevertheless metabolically clustered apart from the wild-type strain. The role of PkpB in glucose and alternative carbon source metabolism remains to be determined (Figure 8). Growth and glucose consumption profiles of the double Δ*pkpA* Δ*pkpB* and Δ*pkpB* Δ*pkpC* strains, also did not shed further light on the role of PkpB in carbon metabolism, as they resembled the parental strains in a condition-dependent manner, suggesting no genetic interaction between *pkpB* and the other two PDHK-encoding genes. In contrast, *pkpA* and *pkpC* genetically interact in the presence of glucose, restoring growth and glucose consumption profiles similar to the wild-type strain, suggesting functional dependencies between these genes. It is possible that the metabolic disturbances caused by the individual deletion of *pkpA* and *pkpC* cancel each other out in the double deletion mutant, thereby restoring glucose utilization profiles. In *S. cerevisiae*, Pkp1p and Pkp2p were shown to physically interact and form heteromers (Krause-Buchholz *et al.* 2006), although they did not phenotypically complement each other, and generation of the corresponding double deletion mutant suggests that they are either part of a kinase cascade or target different proteins in the same pathway (Steensma *et al.* 2008). It will be of interest for future investigations to further unravel the mechanistic nature underlying PkpA and PkpC glucose-mediated metabolic regulation, including determination of PkpA protein interactions as well as further characterizing the aforementioned genetic interactions, especially with regards to strain engineering for biotechnological purposes.

### PkpC is important for hydrolytic enzyme secretion

The aforementioned defects in glucose metabolic utilization in the Δ*pkpC* strain had consequences for protein and protease secretion when grown in CCR conditions (Figure 8A). Cellulase and xylanase activities in the simultaneous presence of glucose and cellulose were much higher than those observed for the wild-type strain in the same conditions. In fact, they were similar to those observed for the wild-type strain when grown solely on cellulose-rich media. PkpC is therefore likely to be involved in hydrolytic enzyme secretion in CCR conditions (Figure 8A), as total amounts of secreted proteins were significantly lower in this strain when compared to the wild-type strain. A major drawback in the conversion of lignocellulosic plant material to biofuels is the CCR-mediated inhibition of enzyme secretion in the presence of glucose in filamentous fungi (Dashtban *et al.* 2009; Mathew *et al.* 2008). The Δ*pkpC* strain is of potential interest for this process, presenting increased secretion of biotechnologically important enzymes and reduced accumulation of biomass, when compared to the wild-type strain in the presence of glucose, although further, additional genetic engineering may be required to generate more effective cellulase and xylanase-secreting strains. Indeed, fold-induction between cellulose and cellulose and glucose condition was similar between the wild-type (∼15-fold) and Δ*pkpC* (∼14-fold) strains and the observed defect in CCR did not increase extracellular enzyme activity in the Δ*pkpC* strain in the presence of glucose and cellulose to the same levels than observed in cellulose-rich conditions only, indicating that CCR is still functional. CCR in *A. nidulans* is a complex process, encompassing different steps and a myriad of proteins such as the scaffold protein CreC of the CreB/CreC de-ubiquitination complex that has been shown to be important for CreA stability (Ries *et al.* 2016); the protein kinases SnfA and PkaA that were shown to be important for CCR through regulating CreA cellular localization by an, as of to date, undefined mechanism (Brown *et al.* 2013; de Assis *et al.* 2015b). In *Neurospora crassa*, VIB1 and COL26 are required for CCR, with the latter involved in repressing *cre-1* expression (Xiong *et al.* 2014). Alternatively, as aforementioned, defects in CCR may only take place during early time points and that after 5 days incubation in cellulose- and glucose-rich media, CCR may be (at least partially) functional again. A similar situation can be envisaged for the Δ*pkpB* strain, which also presented increased extracellular hydrolytic enzyme activity (eightfold and sixfold increase in cellulase and xylanase activity respectively) in the simultaneous presence of glucose and cellulose, although this may likely occur through a different mechanism as total secreted extracellular protein was similar between the Δ*pkpB* and wild-type strains. The impact of the deletion of both PDHK-encoding genes on hydrolytic enzyme secretion and activity is subject to future investigations.

Of further biotechnological interest is the observation that cellulase and xylanase activities were significantly increased in the Δ*pkpA* and Δ*pkpC* strains when compared to the wild-type strain in the presence of cellulose, with both strains presenting a different total protein secretion profile. In addition, PkpA and PkpC appear to be involved specifically in hydrolytic enzyme secretion (Figure 8B), as total secreted protein concentration was lower in the respective deletion strains in the presence of cellulose when compared to the wild-type strain. This is in contrast to (Brown *et al.* 2013), who reported significantly reduced cellulase secretion of the Δ*pkpC* [wrongly annotated as Δ*pkpA* in (Brown *et al.* 2013)] strain. This discrepancy is likely due to the difference in enzyme activity normalization used in both studies (cellulase activity normalization by total intracellular protein used here *vs.* normalization by millilitres). It remains to be determined in what aspects secretion differs in these two strains from the wild-type strain and investigate how these strains are able to increase enzyme secretion, which could occur either at the transcriptional (regulation of cellulase and xylanase-encoding genes) or post-translational (regulation of proteins important for enzyme secretion) level. A link between PDHKs and hydrolytic enzyme secretion has not previously been established in any fungal species, although in *F. graminearum*, a PDHK was shown to be involved in the transcriptional expression of genes encoding the secreted mycotoxin deoxynivalenol (Gao *et al.* 2016), suggesting that PDHKs may be involved in the secretion of different proteomic compounds.

### PkpC regulates the activity of isocitrate lyase (ICL) During growth on acetate

The Δ*pkpC* strain also presented severe growth defects in the presence of the alternative carbon sources cellulose and acetate (Figure 8B, C). This strain was metabolically very different from the wild-type and the other two PDHK deletion strains in the presence of cellulose, with quantitative differences in TCA cycle intermediates and reduced growth phenotypes when grown on precursors feeding into the TCA cycle (Figure 8B). The Δ*pkpC* strain is likely to have impaired use of glucose as well as polysaccharides resulting from cellulose degradation. This would result in starvation, in a de-regulation of central carbon metabolism, as shown by reduced and increased levels of TCA cycle intermediates (Figure 8B) and in a situation that is detrimental to the cell. The exact role of PkpC in cellulose metabolism is subject to future investigations.

Acetate is produced by bacteria of the human gut and lung microbiome or in a tissue-dependent manner, therefore presenting a good carbon source during human host infection by pathogenic fungi (Jiménez-López *et al.* 2013; Borregaard and Herlin 1982; Schug *et al.* 2016; Dickson and Huffnagle 2015; Mirković *et al.* 2015). PkpC was found to potentially interact with and regulate metabolic enzymes required for acetate transport and metabolism, such as the ICL AcuD (Figure 8C). Hyper-activation of AcuD in the the Δ*pkpC* strain may result in a futile glyoxylate cycle therefore blocking acetate utilization, although a defect in acetate uptake cannot be excluded (Figure 8C). These results suggest that PkpC regulates alternative carbon usage through modulating the activity of metabolic enzymes when glucose is absent. In agreement, the cytoplasmic and mitochondrial localization of PkpC in this carbon source allow it to target a wide range of proteins. In *S. cerevisiae*, deletion of the two PDHK-encoding genes *PKP1* and *PKP2*, also resulted in severely reduced growth in the presence of acetate, which was predicted to be based on a futile carbon cycle due to the simultaneous mis-regulation of several metabolic enzymes (Steensma *et al.* 2008). A similar situation may therefore also occur in *A. nidulans*. Furthermore, potential interaction of PkpC with protein kinases involved in different metabolic processes, including the major nutrient sensor and translation-associated protein kinase TorA, may also contribute to the observed growth defects in the presence of glucose, cellulose and acetate. It will be of further interest to study the role of PkpC in acetate usage.

In conclusion, this study identified two PDHKs in *A. nidulans* that integrate carbon source utilization, via metabolic enzyme modulation (Figure 8). Deletion of the corresponding PDHK-encoding genes resulted in a mis-regulation in central carbon metabolism, affecting the secretion of biotechnologically important enzymes and growth on carbon sources that are of importance for the infection process by human pathogenic fungi. This work therefore demonstrates how central carbon metabolism can affect a variety of fungal traits and lays a preliminary basis for further investigation into these processes and traits with the ultimate aim to improve biotechnological applications of fungi or develop novel and improved methods to combat pathogenic fungi.

## References

[bib1] Abad A. J. V. Fernández-Molina J. Bikandi A. Ramírez J. Margareto J., 2010 What makes *Aspergillus fumigatus* a successful pathogen? Genes and molecules involved in invasive aspergillosis. Ver. Iberoam. Micol. 27: 155–182. 10.1016/j.riam.2010.10.003 20974273

[bib2] Al-Bader N. Vanier G. Liu H. Gravelat F. N. Urb M., 2010 Role of trehalose biosynthesis in *Aspergillus fumigatus* development, stress response and virulence. Infect. Immun. 78: 3007–3018. 10.1128/IAI.00813-09 20439478 PMC2897364

[bib3] Amare M. G. Keller N. P., 2014 Molecular mechanisms of *Aspergillus flavus* secondary metabolism and development. Fungal Genet. Biol. 66: 11–18. 10.1016/j.fgb.2014.02.008 24613992

[bib4] Andersen M. R., 2014 Elucidation of primary metabolic pathways in *Aspergillus* species: orphaned research in characterizing orphan genes. Brief. Funct. Genomics 13: 451–455. 10.1093/bfgp/elu029 25114096 PMC4239788

[bib5] Anisimova M. Gascuel O., 2006 Approximate likelihood-ratio test for branches: a fast, accurate and powerful alternative. Syst. Biol. 55: 539–552. 10.1080/10635150600755453 16785212

[bib6] Antoniêto A. C. de Paula R. G. Castro Ldos S. Silva-Rocha R. Persinoti G. F., 2016 *Trichoderma reesei* CRE1-mediated carbon catabolite repression in re-sponse to sophorose through RNA sequencing analysis. Curr. Genomics 17: 119–131. 10.2174/1389202917666151116212901 27226768 PMC4864841

[bib7] Bao H. Kasten S. A. Yan X. Hiromasa Y. Roche T. E., 2004 a Pyruvate dehydrogenase kinase isoform 2 activity stimulated by speeding up the rate of dissociation of ADP. Biochemistry 43: 13442–13451. 10.1021/bi0494875 15491151

[bib8] Bao H. Kasten S. A. Yan X. Roche T. E., 2004 b Pyruvate dehydrogenase kinase isoform 2 activity limited and further inhibited by slowing down the rate of dissociation of ADP. Biochemistry 43: 13432–13441. 10.1021/bi049488x 15491150

[bib9] Blum H. Beier H. Gross H. S., 1987 Improved silver staining of plant proteins, RNA and DNA in polyacrylamide gels. Electrophoresis 8: 93–99. 10.1002/elps.1150080203

[bib10] Borregaard N. Herlin T., 1982 Energy metabolism of human neutrophils during phagocytosis. J. Clin. Invest. 70: 550–557. 10.1172/JCI110647 7107894 PMC370256

[bib11] Bos C. J. Slakhorst M. Visser J. Roberts C. F., 1981 A third unlinked gene controlling the pyruvate dehydrogenase complex in *Aspergillus nidulans*. J. Bacteriol. 148: 594–599. 7028719 10.1128/jb.148.2.594-599.1981 PMC216244

[bib12] Brakhage A. A., 2013 Regulation of fungal secondary metabolism. Nat. Rev. Microbiol. 11: 21–32. 10.1038/nrmicro2916 23178386

[bib13] Brown N. A. de Gouvea P. F. Krohn N. G. Savoldi M. Goldman G. H., 2013 Functional characterisation of the non-essential protein kinases and phosphatases regulating *Aspergillus nidulans* hydrolytic enzyme production. Biotechnol. Biofuels 6: 91 10.1186/1754-6834-6-91 23800192 PMC3698209

[bib14] Chen F. Mackey A. J. Jr. Stoeckert C. J. Roos D. S., 2006 OrthoMCL-DB: querying a comprehensive multi-species collection of ortholog groups. Nucleic Acids Res. 34: D363–D368. 10.1093/nar/gkj123 16381887 PMC1347485

[bib15] Chretien D. Pourrier M. Bourgeron T. Séné M. Rötig A., 1995 An improved spectrophotometric assay of pyruvate dehydrogenase in lactate dehydrogenase contaminated mitochondrial preparations from human skeletal muscle. Clin. Chim. Acta 240: 129–136. 10.1016/0009-8981(95)06145-6 8548923

[bib16] Claros M. G. Vincens P., 1996 Computational method to predict mitochondrially imported proteins and their targeting sequences. Eur. J. Biochem. 241: 779–786. 10.1111/j.1432-1033.1996.00779.x 8944766

[bib17] Costenoble R. Picotti P. Reiter L. Stallmach R. Heinemann M., 2011 Comprehensive quantitative analysis of central carbon and amino-acid metabolism in *Saccharomyces cerevisiae* under multiple conditions by targeted proteomics. Mol. Syst. Biol. 7: 464 10.1038/msb.2010.122 21283140 PMC3063691

[bib18] Creaser E. H. Porter R. L. Britt K. A. Pateman J. A. Doy C. H., 1985 Purification and preliminary characterization of alcohol dehydrogenase from *Aspergillus nidulans*. Biochem. J. 225: 449–454. 10.1042/bj2250449 3156582 PMC1144610

[bib19] Cuadros-Inostroza A. Caldana C. Redestig H. Kusano M. Lisec J., 2009 TargetSearch - a Bioconductor package for the efficient preprocessing of GC-MS metabolite profiling data. BMC Bioinformatics 10: 428 10.1186/1471-2105-10-428 20015393 PMC3087348

[bib20] Dashtban M. Schraft H. Qin W., 2009 Fungal bioconversion of lignocellulosic residues; opportunities and perspectives. Int. J. Biol. Sci. 5: 578–595. 10.7150/ijbs.5.578 19774110 PMC2748470

[bib21] de Assis L. J. Ries L. N. A. Savoldi M. Dinamarco T. M. Goldman G. H., 2015 a Multiple phosphatases regulate carbon source-dependent germination and primary metabolism in *Aspergillus nidulans*. G3 (Bethesda) 5: 857–872. 10.1534/g3.115.016667 25762568 PMC4426372

[bib22] de Assis L. J. Ries L. N. A. Savoldi M. Dos Reis T. F. Brown N. A., 2015 b *Aspergillus nidulans* protein kinase A plays an important role in cellulase production. Biotechnol. Biofuels 8: 213 10.1186/s13068-015-0401-1 26690721 PMC4683954

[bib23] de Castro Pimentel Figueiredo B. de Castro P. A. Dinamarco T. M. Goldman M. H. Goldman G. H., 2011 The *Aspergillus nidulans nucA*(EndoG) homologue is not involved in cell death. Eukaryot. Cell 10: 276–283. 10.1128/EC.00224-10 21131437 PMC3067401

[bib24] De Souza C. P. Hashmi S. B. Osmani A. H. Andrews P. Ringelberg C. S., 2013 Functional analysis of the *Aspergillus nidulans* kinome. PLoS One 8: e58008 10.1371/journal.pone.0058008 23505451 PMC3591445

[bib25] Dereeper A. Audic S. Claverie J. M. Blanc G., 2010 BLAST-EXPLORER helps you building datasets for phylogenetic analysis. BMC Evol. Bio. 10 10.1186/1471-2148-10-8 PMC2821324 20067610

[bib26] Dereeper A. Guignon V. Blanc G. Audic S. Buffet S., 2008 Phylogeny.fr: robust phylogenetic analysis for the non-specialist. Nucleic Acids Res. 36: W465–W469. 10.1093/nar/gkn180 18424797 PMC2447785

[bib27] Dickson R. P. Huffnagle G. B., 2015 The lung microbiome: new principles for respiratory bacteriology in health and disease. PLoS Pathog. 11: e1004923 10.1371/journal.ppat.1004923 26158874 PMC4497592

[bib28] Emanuelsson O. Nielsen H. Brunak S. von Heijne G., 2000 Predicting subcellular localization of proteins based on their N-terminal amino acid sequence. J. Mol. Biol. 300: 1005–1016. 10.1006/jmbi.2000.3903 10891285

[bib29] Fernandez J. Wright J. D. Hartline D. Quispe C. F. Madayiputhiya N., 2012 Principles of carbon catabolite repression in the rice blast fungus: Tps1, Nmr1–3, and a MATE-family pump regulate glucose metabolism during infection. PLoS Genet. 8: e1002673 10.1371/journal.pgen.1002673 22570632 PMC3342947

[bib30] Fleck C. B. Brock M., 2010 *Aspergillus fumigatus* catalytic glucokinase and hexokinase: expression analysis and importance for germination, growth, and conidiation. Eukaryot. Cell 9: 1120–1135. 10.1128/EC.00362-09 20453072 PMC2901669

[bib31] Gao T. Chen J. Shi Z., 2016 *Fusarium graminearum* pyruvate dehydrogenase kinase 1 is critical for conidiation, mycelium growth and pathogenicity. PLoS One. 10.1371/journal.pone.0158077 PMC4920349 27341107

[bib32] Gey U. Czupella C. Hoflack B. Rödel G. Krause-Buchholz U., 2008 Yeast pyruvate dehydrogenase complex is regulated by a concerted activity of two kinases and two phosphatases. J. Biol. Chem. 283: 9759–9767. 10.1074/jbc.M708779200 18180296

[bib33] Giavalisco P. Li Y. Matthes A. Eckhardt A. Hubberten H. M., 2011 Elemental formula annotation of polar and lipophilic metabolites using (13)C, (15)N and (34)S isotope labelling, in combination with high- resolution mass spectrometry. Plant J. 68: 364–376. 10.1111/j.1365-313X.2011.04682.x 21699588

[bib34] Gietz R. D. Schiestl R. H., 1991 Applications of high efficiency lithium acetate transformation of intact yeast cells using single-stranded nucleic acids as carrier. Yeast 7: 253–263. 10.1002/yea.320070307 1882550

[bib35] Goldman G. H. dos Reis Marques E. Duarte Ribeiro D. C. de Souza Bernardes L. A. Quiapin A. C., 2003 Expressed sequence tag analysis of the human pathogen *Paracoccidioides brasiliensis* yeast phase: identification of putative homologues of *Candida albicans* virulence and pathogenicity genes. Eukaryot. Cell 2: 34–48. 10.1128/EC.2.1.34-48.2003 12582121 PMC141168

[bib36] Grahl N. Puttikamonkul S. Macdonald J. M. Gamcsik M. P. Ngo L. Y., 2011 *In vivo* hypoxia and a fungal alcohol dehydrogenase influence the pathogenesis of invasive pulmonary aspergillosis. PLoS Pathog. 7: e1002145 10.1371/journal.ppat.1002145 21811407 PMC3141044

[bib37] Habelhah H. Laine A. Erdjument-Bromage H. Tempst P. Gershwin M. E., 2004 Regulation of 2-oxoglutarate (alpha-ketoglutarate) dehydrogenase stability by the RING finger ubiquitin ligase Siah. J. Biol. Chem. 279: 53782–53788. 10.1074/jbc.M410315200 15466852

[bib38] Hayer K. Stratford M. Archer D. B., 2013 Structural features of sugars that trigger or support conidial germination in the filamentous fungus *Aspergillus niger*. Appl. Environ. Microbiol. 79: 6924–6931. 10.1128/AEM.01078-14 23995938 PMC3811532

[bib39] Hondmann D. H. A. Busink R. Witteveen C. F. Visser J. 1991 Glycerol catabolism in *Aspergillus nidulans*. J. Gen. Microbiol. 137: 629–636. 10.1099/00221287-137-3-629 2033381

[bib40] Huege J. Krall L. Steinhauser M. C. Giavalisco P. Rippka R., 2011 Sample amount alternatives for data adjustment in comparative cyanobacterial metabolomics. Anal. Bioanal. Chem. 399: 3503–3517. 10.1007/s00216-011-4678-z 21340691

[bib41] Hynes M. J. Murray S. L., 2010 ATP-citrate lyase is required for production of cytosolic acetyl coenzyme A and development in *Aspergillus nidulans*. Eukaryot. Cell 9: 1039–1048. 10.1128/EC.00080-10 20495057 PMC2901662

[bib42] Hynes M. J. Murray S. L. Duncan A. Khew G. S. Davis M. A., 2006 Regulatory genes controlling fatty acid catabolism and peroxisomal functions in the filamentous fungus *Aspergillus nidulans*. Eukaryot. Cell 5: 794–805. 10.1128/EC.5.5.794-805.2006 16682457 PMC1459687

[bib43] Inglis D. O. Binkley J. Skrzypek M. S. Arnaud M. B. Cerqueira G. C., 2013 Comprehensive annotation of secondary metabolite biosynthetic genes and gene clusters of *Aspergillus nidulans*, *A. fumigatus*, *A. niger* and *A. oryzae*. BMC Microbiol. 13: 91 10.1186/1471-2180-13-91 23617571 PMC3689640

[bib44] Jiménez-López C. Collette J. R. Brothers K. M. Shepardson K. M. Cramer R. A., 2013 *Candida albicans* induces arginine biosynthetic genes in response to host-derived reactive oxygen species. Eukaryot. Cell 12: 91–100. 10.1128/EC.00290-12 23143683 PMC3535846

[bib45] Klejnstrup M. L. Frandsen R. J. Holm D. K. Nielsen M. T. Mortensen U. H., 2012 Genetics of polyketide metabolism in *Aspergillus nidulans*. Metabolites 2: 100–133. 10.3390/metabo2010100 24957370 PMC3901194

[bib46] Kolobova E. Tuganova A. Boulatnikov I. Popov K. M., 2001 Regulation of pyruvate dehydrogenase activity through phosphorylation at multiple sites. J. Biochem. 358: 69–77. 10.1042/bj3580069 PMC1222033 11485553

[bib47] Krause-Buchholz U. Gey U. Wünschmann J. Becker S. Rödel G., 2006 *YIL042c* and *YOR090c* encode the kinase and phosphatase of the *Saccharomyces cervisiae* pyruvate dehydrogenase complex. FEBS Lett. 580: 2553–2560. 10.1016/j.febslet.2006.04.002 16643908

[bib48] Letunic I. Doerks T. Bork P., 2015 SMART: recent updates, new developments and status in 2015. Nucleic Acids Res. 43: D257–D260. 10.1093/nar/gku949 25300481 PMC4384020

[bib50] Mathew G. M. Sukumaran R. K. Singhania R. R. Pandey A., 2008 Progress in research on fungal cellulases for lignocelluloses degradation. J. Sci. Ind. Res. (India) 67: 898–907.

[bib51] Mirković B. Murray M. A. Lavelle G. M. Molloy K. Azim A. A., 2015 The role of short-chain fatty acids, produced by anaerobic bacteria, in the cystic fibrosis airway. Am. J. Respir. Crit. Care Med. 192: 1314–1324. 10.1164/rccm.201505-0943OC 26266556 PMC4731701

[bib52] Nayak T. E. Szewczyk C. E. Oakley A. Osmani L. Ukil L., 2006 A versatile and efficient gene-targeting system for *Aspergillus nidulans*. Genetics 172: 1557–1566. 10.1534/genetics.105.052563 16387870 PMC1456264

[bib53] Niu J. T. G. Homan M. Arentshorst R. P. de Vries J. Visser J., 2015 The interaction of induction and repression mechanisms in the regulation of galacturonic acid-induced genes in *Aspergillus niger*. Fungal Genet. Biol. 82: 32–42. 10.1016/j.fgb.2015.06.006 26127014

[bib54] Patel M. S. Korotchkina L. G., 2006 Regulation of the pyruvate dehydrogenase complex. Biochem. Soc. Trans. 34: 217–222. 10.1042/BST0340217 16545080

[bib55] Payton M. A. Mc Cullough W. Roberts C. F. Guest J. R., 1977 Two unlinked genes for the pyruvate dehydrogenase complex in *Aspergillus nidulans*. J. Bacteriol. 129: 1222–1226. 321417 10.1128/jb.129.3.1222-1226.1977 PMC235084

[bib56] Ries L. N. A. Beattie S. R. Espeso E. A. Cramer R. A. Goldman G. H., 2016 Diverse regulation of the CreA carbon catabolite repressor in *Aspergillus nidulans*. Genetics 203: 335–352. 10.1534/genetics.116.187872 27017621 PMC4858783

[bib57] Roessner U. Luedemann A. Brust D. Fiehn O. Linke T., 2001 Metabolic profiling allows comprehensive phenotyping of genetically or environmentally modified plant systems. Plant Cell 13: 11–29. 10.1105/tpc.13.1.11 11158526 PMC2652711

[bib58] Rohde J. R. Bastidas R. Puria R. Cardenas M. E., 2008 Nutritional control via Tor signalling in *Saccharomyces cerevisiae*. Curr. Opin. Microbiol. 11: 153–160. 10.1016/j.mib.2008.02.013 18396450 PMC2394285

[bib59] Ruijter G. J. Visser J., 1997 Carbon repression in Aspergilli. FEMS Microbiol. Lett. 151: 103–114. 10.1111/j.1574-6968.1997.tb12557.x 9228741

[bib60] Sambrook J. Russell D. W., 2001 Molecular Cloning: A Laboratory Manual, Ed. 4th Cold Spring Harbor Laboratory Press, Cold Spring Harbor, NY.

[bib61] Schug Z. T. Vande Voorde J. Gottlieb E., 2016 The metabolic fate of acetate in cancer. Nat. Rev. Cancer 16: 708–717. 10.1038/nrc.2016.87 27562461 PMC8992383

[bib62] Schultz J. Milpetz F. Bork P. Ponting C. P., 1998 SMART, a simple modular architecture research tool: identification of signaling domains. Proc. Natl. Acad. Sci. USA 95: 5857–5864. 10.1073/pnas.95.11.5857 9600884 PMC34487

[bib63] Shimizu M. Fujii T. Masuo S. Takaya N., 2010 Mechanism of *de novo* branched-chain amino acid synthesis as an alternative electron sink in hypoxic *Aspergillus nidulans* cells. Appl. Environ. Microbiol. 76: 1507–1515. 10.1128/AEM.02135-09 20081005 PMC2832390

[bib64] Stacklies W. Redestig H. Scholz M. Walther D. Selbig J., 2007 pcaMethods–a bioconductor package providing PCA methods for incomplete data. Bioinformatics 23: 1164–1167. 10.1093/bioinformatics/btm069 17344241

[bib65] Steensma H. Y. Tomaska L. Reuven P. Nosek J. Brandt R., 2008 Disruption of genes encoding pyruvate dehydrogenase kinases leads to retarded growth on acetate and ethanol in *Saccharomyces cerevisiae*. Yeast 25: 9–19. 10.1002/yea.1543 17918780

[bib66] Sun J. Glass N. L., 2016 Identification of the CRE-1 cellulolytic regulon in *Neurospora crassa*. PLoS One 6 10.1371/journal.pone.0025654 PMC3183063 21980519

[bib67] Thevelein J. M., 1984 Regulation of trehalose mobilisation in fungi. Microbiol. Rev. 48: 42–59. 6325857 10.1128/mr.48.1.42-59.1984 PMC373002

[bib68] Tretter L. Adam-Vizi V., 2004 Generation of reactive oxygen species in the reaction catalyzed by alpha-ketoglutarate dehydrogenase. J. Neurosci. 24: 7771–7778. 10.1523/JNEUROSCI.1842-04.2004 15356188 PMC6729921

[bib69] Vödisch M. Albrecht D. Lessing F. Schmidt A. D. Winkler R., 2009 Two-dimensional proteome reference maps for the human pathogenic filamentous fungus *Aspergillus fumigatus*. Proteomics 9: 1407–1415. 10.1002/pmic.200800394 19253289

[bib70] Weckwerth W. Wenzel K. Fiehn O., 2004 Process for the integrated extraction, identification and quantification of metabolites, proteins and RNA to reveal their co-regulation in biochemical networks. Proteomics 4: 78–83. 10.1002/pmic.200200500 14730673

[bib71] Wieland O. H. Hartmann U. Siess E. A., 1972 *Neurospora crassa* pyruvate dehydrogenase: interconversion by phosphorylation and dephosphorylation. FEBS Lett. 27: 240–244. 10.1016/0014-5793(72)80630-6 4352021

[bib72] Wu P. Peters J. M. Harris R. A., 2001 Adaptive increase in pyruvate dehydrogenase kinase 4 during starvation is mediated by peroxisome proliferator-activated receptor alpha. Biochem. Biophys. Res. Commun. 287: 391–396. 10.1006/bbrc.2001.5608 11554740

[bib73] Xiong Y. Sun J. Glass N. L., 2014 VIB1, a link between glucose signalling and carbon catabolite repression, is essential for plant cell wall degradation by *Neurospora crassa*. PLoS Genet. 10: e1004500 10.1371/journal.pgen.1004500 25144221 PMC4140635

